# MARCH2-mediated Lys63-linked polyubiquitination promotes metastasis by modulating the catalytic activity of TGF-β type I receptor

**DOI:** 10.1038/s41419-025-08145-3

**Published:** 2025-11-10

**Authors:** Kun Tae, Sang Woo Cho, Seonjeong Lee, Dahyoon Heo, Hyo Sun Cha, Da Yeon Lee, Eunjeong Oh, Minhyeong Choi, Donghyuk Shin, Siyoung Yang, Cheolju Lee, Cheol Yong Choi

**Affiliations:** 1 Department of Biological Sciences, Sungkyunkwan University, Suwon, Republic of Korea; 2 Chemical & Biological Integrative Research Center, Korea Institute of Science and Technology, Seoul, Republic of Korea; 3 Department of Systems Biology, College of Life Science and Biotechnology, Yonsei University, Seoul, Republic of Korea; 4 Division of Bio-Medical Science & Technology, KIST school, University of Science and Technology, Seoul, Republic of Korea

**Keywords:** Kinases, Growth factor signalling

## Abstract

The TGF-β signaling pathway is initiated when the type II receptor phosphorylates the type I receptor (ALK5) upon TGF-β binding. While E3 ubiquitin ligases regulate TGF-β receptor degradation, their role in modulating receptor catalytic activity via ubiquitination remains largely unexplored. Here, we demonstrate that the E3 ubiquitin ligase MARCH2 enhances ALK5 catalytic activity by conjugating K63-linked ubiquitin chains to lysines 342/343 (K342/343), primarily at endosomes following TGF-β-induced endocytosis. Mutations of ALK5 at K342/343 (K342/343R) abolish its catalytic activity for SMAD2 phosphorylation, leading to impaired TGF-β responses and reduced cell migration in A549 cells. In a mouse model, expression of the ALK5 K342/343 R mutant significantly decreases lung metastasis compared to wild-type ALK5. TCGA analysis further revealed a strong positive correlation between MARCH2 expression and TGF-β target gene expression. Collectively, these findings establish ALK5 ubiquitination at K342/343 by MARCH2 as a crucial regulatory mechanism for ALK5 catalytic activity, TGF-β signaling, and metastasis.

## Introduction

Membrane-Associated RING-CH (MARCH) proteins constitute a family of eleven members (MARCH1-11), initially identified through bioinformatics analyses of mammalian homologs of the K3 and K5 E3 ligases encoded by Kaposi’s sarcoma-associated herpesvirus (KSHV) [1–3]. Most MARCH family proteins share a conserved structure, comprised of an N-terminal RING finger domain followed by C-terminal transmembrane domains (TMs) [4, 5]. Phylogenetic analyses classify MARCH proteins into five subgroups, with MARCH2 exhibiting the highest sequence similarity to MARCH3 [5]. Notably, the non-canonical members MARCH7 and MARCH10 lack transmembrane domains, distinguishing them from other family members [4, 5]. Structurally, MARCH2 contains an N-terminal zinc ion-associated RING domain, which plays a crucial role in recognizing substrates and interacting with cognate E2 ubiquitin-conjugating enzymes [6]. Additionally, MARCH2 possesses two hydrophobic helical transmembrane domains (TM1 and TM2) containing aligned hydrophobic amino acids and GxxxG motif variants (AxxxG in TM1 and AxxxA in TM2), which may facilitate dimerization or substrate recognition [5]. A PDZ-binding motif at the extreme C-terminus regulates interactions with partner proteins and intracellular localization [7, 8].

MARCH2 participates in diverse cellular processes, including immune regulation, antiviral defense, autophagy, and tumor progression, primarily through the ubiquitination and subsequent degradation of membrane-associated and cytosolic target proteins [9–12]. In immune regulation, MARCH2 modulates surface receptor levels and signaling proteins, reducing ligand binding and impairing viral production by targeting viral envelope proteins [13]. MARCH2 negatively regulates NF-κB and type I interferon (IFN-I) signaling by promoting K48-linked ubiquitination and degradation of NEMO [14]. Additionally, MARCH2 downregulates interleukin-5 receptor α (IL-5Rα), thereby mitigating allergic airway inflammation [15], and regulates interleukin-6 receptor α (IL6-Rα) redundantly with MARCH3 and MARCH4 [16]. In tumor progression, MARCH2 plays context-dependent roles [17]. MARCH2 is upregulated in hepatocellular carcinoma and colorectal cancer, promoting invasion, migration, and epithelial-mesenchymal transition (EMT) [18, 19]. In contrast, in triple-negative breast cancer (TNBC), MARCH2 functions as a tumor suppressor. Loss of PTK6, a kinase that stabilizes Snail to promote EMT, enhances MARCH2-mediated ubiquitination and degradation of Snail, thereby reversing mesenchymal traits and suppressing TNBC metastasis [20]. While many MARCH2 substrates undergo degradation following ubiquitination, some targets are not degraded. For example, MARCH2 ubiquitinates Ebola virus glycoproteins, retaining them in the trans-Golgi network (TGN) and preventing their translocation to the plasma membrane [21]. Similarly, DLG1 is ubiquitinated and colocalized with MARCH2 at cell-cell contact sites without significant changes in protein abundance [8]. These findings highlight the substrate-specific outcomes of MARCH2-mediated ubiquitination. Most MARCH2 substrates are ubiquitinated via K48- or K27-linked chains. For instance, NEMO and PGAM5 undergo K48-linked ubiquitination for proteasomal degradation [14, 22], whereas IKKε and IL-5Rα are modified with K27-linked ubiquitin for lysosomal degradation [15, 23]. Collectively, MARCH2 plays a crucial role in immune escape, antiviral defense, inflammation, cellular trafficking, and tumor progression through both degradable and non-degradable ubiquitination.

The transforming growth factor-β (TGF-β) signaling pathway regulates numerous cellular processes, including embryonic development, cell proliferation, differentiation, migration, immune responses, and homeostasis [24, 25]. Notably, TGF-β signaling exhibits a dual role in tumor progression, functioning as a tumor suppressor in normal cells and early-stage carcinomas, but promoting tumor progression, invasion, and metastasis in advanced tumors [26, 27]. Dysregulation of TGF-β signaling is implicated in various diseases, including developmental defects, fibrosis, infectious disease, inflammatory conditions, and cancer metastasis [28]. TGF-β signaling is initiated when a ligand binds to the TGF-β type II receptor (TGFBR2), which recruits and heterotetramerizes with the type I receptor (TGFBR1 or ALK5). Within this complex, the constitutively active type II receptor phosphorylates the glycine-serine (GS)-rich domain of the type I receptor, inducing a conformational change that causes catalytic activation [29]. The phosphorylated GS domain serves as a docking site for receptor-regulated SMAD proteins, primarily SMAD2 and SMAD3, which are subsequently phosphorylated by the activated type I receptor. Phosphorylated SMAD2/3 forms a complex with SMAD4, which translocates to the nucleus to regulate the transcription of TGF-β target genes [30]. While receptor phosphorylation is essential for TGF-β pathway activation, ubiquitination plays a crucial role in regulating TGF-β receptor stability and function. The inhibitory SMAD protein SMAD7, induced by TGF-β signaling, recruits E3 ubiquitin ligases such as SMURF1, SMURF2, NEDD4-2, and WWP1 to the TGF-β receptors, leading to K48-linked ubiquitination and proteasomal degradation of type I and II receptors [31]. This negative feedback mechanism fine-tunes the intensity and duration of TGF-β signaling by modulating receptor levels. In contrast, TRAF6-mediated K63-linked ubiquitination of ALK5 facilitates its proteolytic cleavage via TNF-α converting enzyme (TACE) and presenilin-1 [32]. The cleaved intracellular domain (ICD) translocates to the nucleus, where it promotes target gene expression to enhance cell invasiveness [33].

In this study, we identify MARCH2 as a novel regulator of ALK5 ubiquitination and catalytic function. Using mass spectrometry, we discovered that MARCH2 interacts with ALK5 in a TGF-β-dependent manner and mediates its ubiquitination at multiple lysine residues through K27- and K63-linked ubiquitin chains. Notably, we demonstrate that K63-linked ubiquitination at lysines 342/343 (K342/343) enhances the catalytic activity of ALK5. Functionally, a K342/343 R substitution impairs TGF-β-induced cell migration, invasion, and metastasis in a mouse model. These findings establish MARCH2 as a critical homeostatic regulator of TGF-β signaling and highlight its potential role in modulating tumor metastasis.

## Results

### MARCH2 regulates the TGF-β signaling pathway

**Fig. 1:**
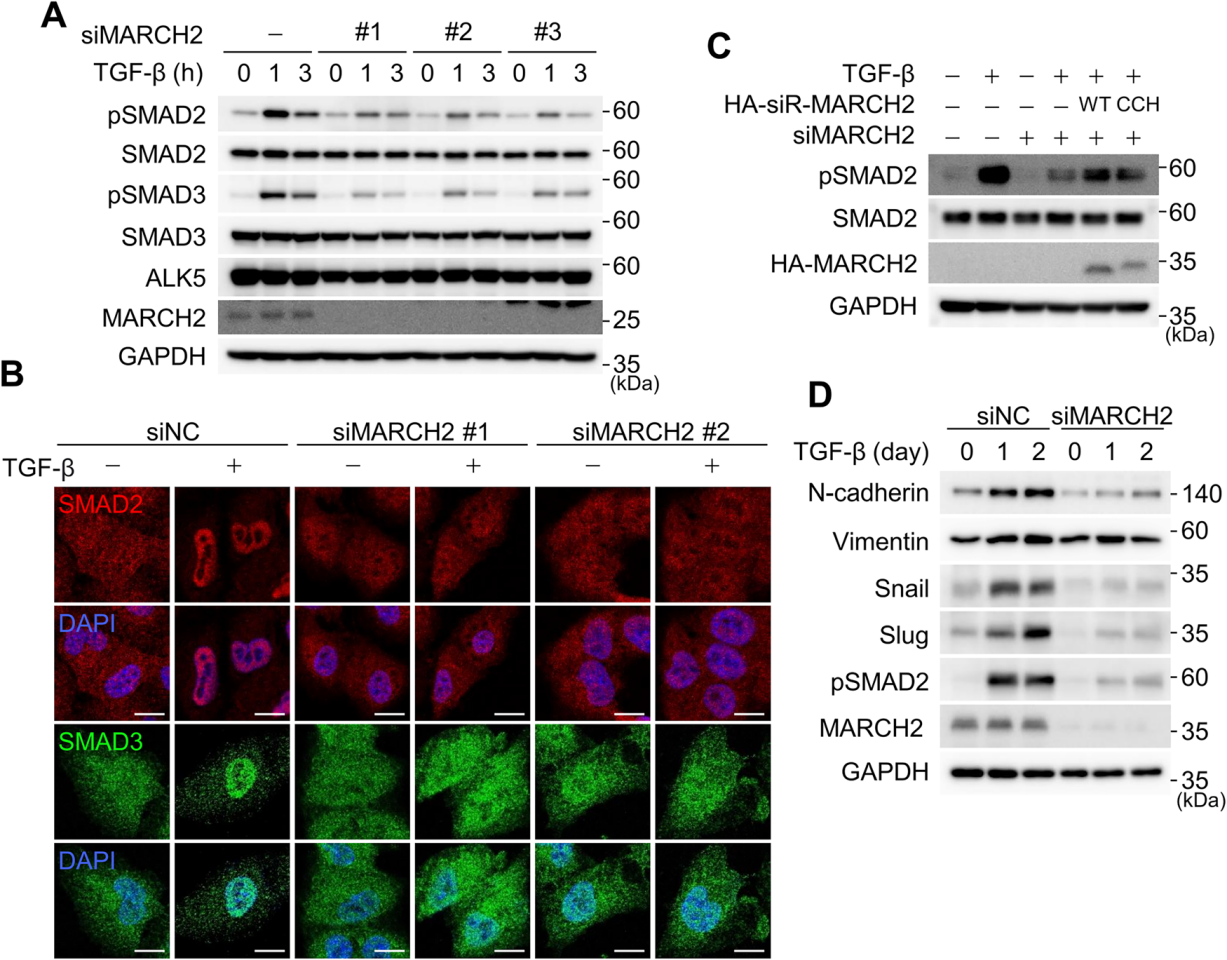
MARCH2 regulates TGF-β signaling in an E3 ligase-dependent manner. **A** A549 cells were transfected with three distinct siRNAs targeting MARCH2 and subsequently treated with TGF-β for 1 or 3 h. Activation of TGF-β signaling was assessed by immunoblotting with the indicated antibodies. **B** A549 cells transfected with MARCH2-targeting siRNAs were treated with TGF-β for 1 h and immunostained with anti-SMAD2 and anti-SMAD3 antibodies. Nuclei were counterstained with DAPI. Scale bar: 10 μm. **C** A549 cells transfected with the indicated siRNAs were infected with a lentivirus expressing siRNA-resistant HA-MARCH2 (HA-siR-MARCH2) or a catalytically inactive mutant. The cells were then treated with TGF-β for 1 h. CCH denotes a catalytically inactive MARCH2 mutant. **D** A549 cells transfected with the indicated siRNAs were treated with TGF-β for 1 or 2 days and analyzed by immunoblotting with the indicated antibodies. Uncropped full-length blots are presented in Supplementary Fig. 1.

MARCH2 has been implicated in invasion, migration, and epithelial-mesenchymal transition (EMT) in hepatocellular carcinoma (HCC) and colorectal cancer cells [18, 19]. However, its molecular targets and the signaling pathways underlying MARCH2-induced cell migration and EMT remain largely unexplored. Given that the TGF-β signaling pathway is a key driver of cell migration and EMT, we investigated whether MARCH2 plays a role in TGF-β signaling. In A549 cells, TGF-β treatment induced SMAD2/3 phosphorylation, but this response was diminished in MARCH2-depleted cells. The use of three independent siRNAs targeting MARCH2 yielded consistent results, ruling out off-target effects (Fig. 1A). To further assess the impact of MARCH2 depletion on TGF-β signaling, we examined the nuclear translocations of SMAD2 and SMAD3. In TGF-β-treated A549 cells, SMAD2 and SMAD3 translocated to the nucleus, but this translocation was inhibited upon MARCH2 depletion (Fig. 1B). To determine whether the E3 ligase activity of MARCH2 is required for TGF-β signaling, we tested a catalytically inactive MARCH2 mutant in which cysteine and histidine residues (C64S, C67S, and H90Q) were substituted. While MARCH2 depletion reduced SMAD2 phosphorylation, this effect was rescued by siRNA-resistant wild-type MARCH2 but not by the catalytically inactive mutant, indicating that the E3 ubiquitin ligase activity of MARCH2 is essential for SMAD2 phosphorylation (Fig. 1C). Furthermore, MARCH2 depletion resulted in reduced expression of TGF-β target genes, including Snail, Slug, N-cadherin, and Vimentin, suggesting that MARCH2 facilitates TGF-β signaling at the level of SMAD2 phosphorylation (Fig. 1D).

### MARCH2 interacts with the TGF-β type I receptor via both the transmembrane and cytoplasmic domains

**Fig. 2:**
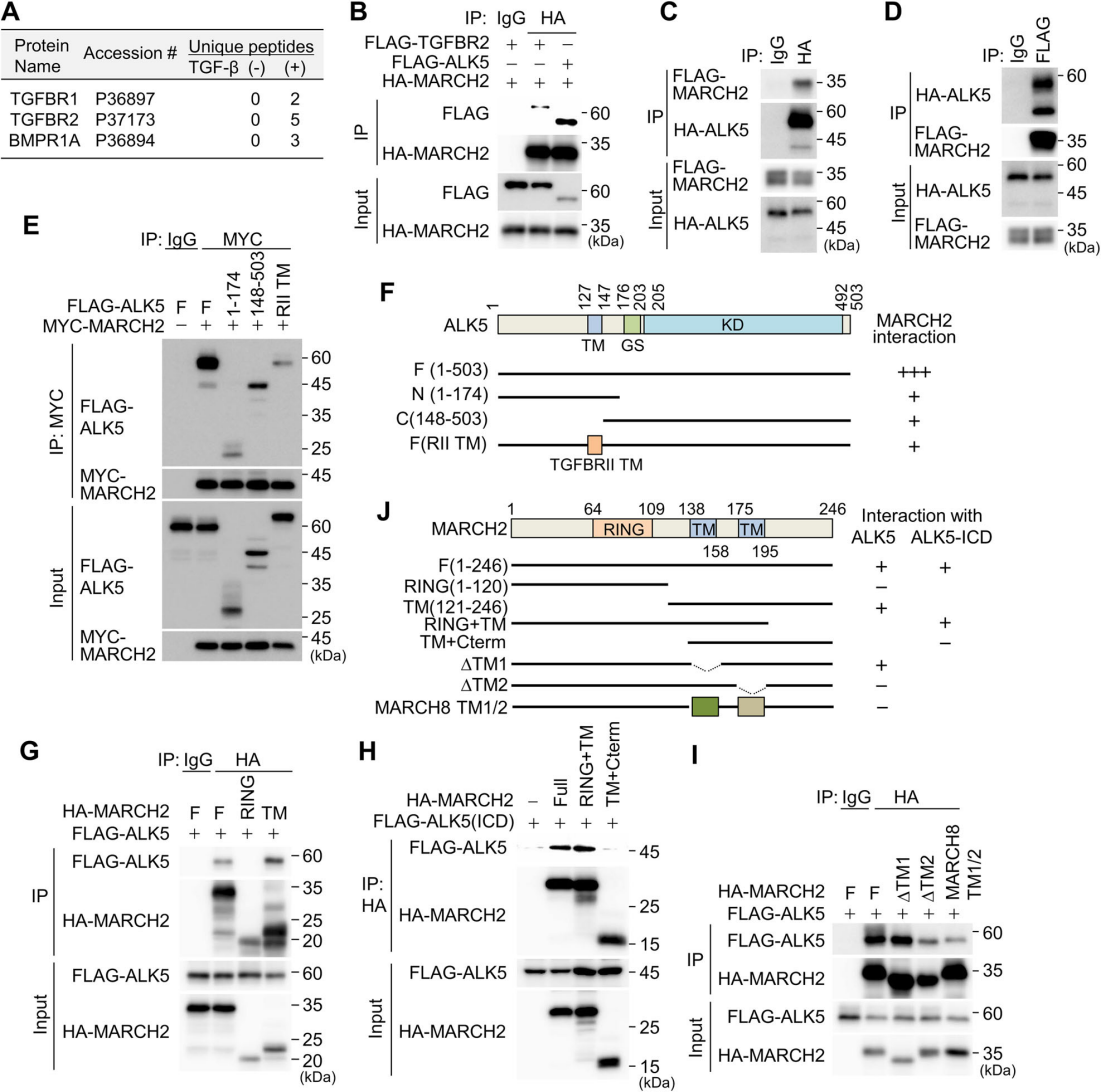
Interaction of MARCH2 and ALK5 occurs through the transmembrane and cytoplasmic domains. **A** MARCH2-interacting proteins were identified via mass spectrometry. Proteins involved in TGF-β signaling are shown. TGF-β receptors and BMP receptor type 1 A were detected exclusively under TGF-β treatment conditions. **B** A co-immunoprecipitation (co-IP) assay was performed to analyze the interaction of HA-MARCH2 with FLAG-ALK5 or FLAG-TGFBR2 in HEK293T cells. HA-MARCH2 was immunoprecipitated using an HA antibody, and co-precipitated proteins were analyzed by immunoblotting with the indicated antibodies. **C** A co-IP assay was conducted to assess the interaction between HA-ALK5 and FLAG-MARCH2. Proteins co-precipitated with HA-ALK5 were detected by immunoblotting with the indicated antibodies. **D** A co-IP assay was performed to examine the interaction between FLAG-MARCH2 and HA-ALK5. Proteins co-precipitated with FLAG-MARCH2 were analyzed by immunoblotting with the indicated antibodies. **E** The ALK5 domain mediating interaction with MARCH2 was analyzed using a co-IP assay. Proteins co-precipitated with MYC-MARCH2 were detected by immunoblotting with the indicated antibodies. **F** A schematic representation of ALK5 domains and deletion mutants. Symbols “+” and “+++” indicate low and strong binding affinities with MARCH2, respectively. **G** The MARCH2 domain mediating interaction with ALK5 was analyzed via a co-IP assay. Proteins co-precipitated with HA-MARCH2 were detected by immunoblotting with the indicated antibodies. **H** A co-IP assay was conducted to evaluate the interaction of FLAG-ALK5 ICD (148-503) with HA-MARCH2 or its deletion mutants. Proteins co-precipitated with HA-MARCH2 were analyzed by immunoblotting with the indicated antibodies. **I** A co-IP assay was conducted to compare the interaction of FLAG-ALK5 with HA-MARCH2 or an HA-MARCH2 TM1/2-swapped mutant. HA-MARCH8 TM1/2 refers to a mutant in which TM1 and TM2 of MARCH2 were replaced with those of MARCH8. **J** A schematic representation of MARCH2 domain organization and its deletion mutants. “+” indicates interaction between MARCH2 and ALK5 in the co-IP assay.

To identify MARCH2 binding partners involved in TGF-β signaling, we generated an A549 cell line expressing HA-MARCH2 and isolated HA-MARCH2-interacting proteins under both TGF-β treated and untreated conditions. Mass spectrometry analysis revealed the TGF-β type I and II receptors as MARCH2-interacting proteins (Fig. 2A and Table S1). Notably, these interactions were detected only in TGF-β-treated samples, indicating that TGF-β signaling promotes MARCH2 binding to these receptors. To validate these interactions, we performed co-immunoprecipitation (co-IP) assays. Flag-tagged TGF-β type I receptor (ALK5) efficiently co-precipitated with HA-MARCH2, whereas the TGF-β type II receptor exhibited a relatively lower affinity for MARCH2 (Fig. 2B). Given that the TGF-β type II receptor was not ubiquitinated by MARCH2 (Fig. S1A), we focused subsequent studies on the MARCH2-ALK5 interaction. The interaction between MARCH2 and ALK5 was confirmed by co-IP assays (Fig. 2C, D) and further validated in live cells using a bimolecular fluorescence complementation (BiFC) assay (Fig. S1B). To further characterize the MARCH2-ALK5 interaction, we conducted domain mapping using serial deletion mutants. ALK5 domain analysis revealed that full-length ALK5 binds strongly to MARCH2, while the transmembrane (TM)-containing N-terminus and the cytoplasmic C-terminus alone exhibit weaker binding (Fig. 2E). To test the importance of the TM domain for MARCH2 binding, we replaced the ALK5 TM domain with that of the TGF-β type II receptor, which significantly reduced MARCH2 binding compared to binding with full-length wild-type ALK5. This suggests that both the TM and cytoplasmic domains of ALK5 are required for efficient MARCH2 recognition (Fig. 2E, F). Similarly, domain analysis of MARCH2 revealed that the TM-containing C-terminus, but not the RING domain alone, contributed to full-length ALK5 binding (Fig. 2G). Since the intracellular domain (ICD; aa 148-503) of ALK5 was found to interact with MARCH2 (Fig. 2E), we used ALK5 ICD for co-IP assays. Immunoblotting revealed that the MARCH2 RING domain plus TM domains, but not the C-terminus plus TM domains, interact with ALK5 ICD (Fig. 2H), suggesting that the MARCH2 RING domain interacts with the ALK5 ICD. To further investigate the role of the MARCH2 TM domains in ALK5 binding, we generated deletion constructs removing either TM1 or TM2, along with a construct where both TM1 and TM2 were swapped with the TMs of MARCH8, a negative control that does not interact with ALK5 (Fig. S1C). Co-IP experiments showed that deleting TM2, but not TM1, or swapping both TM1/2, significantly reduced ALK5 binding (Fig. 2I). These findings demonstrate that both MARCH2 TM2 and the RING domain contribute to ALK5 binding (Fig. 2J). Taken together, these results indicate that MARCH2 preferentially interacts with ALK5 rather than the TGF-β type II receptor. This interaction involves the TM domains of both proteins, as well as their cytoplasmic regions, specifically the MARCH2 RING domain and the ALK5 ICD.

### MARCH2 interacts with ALK5 at the early endosomes

**Fig. 3:**
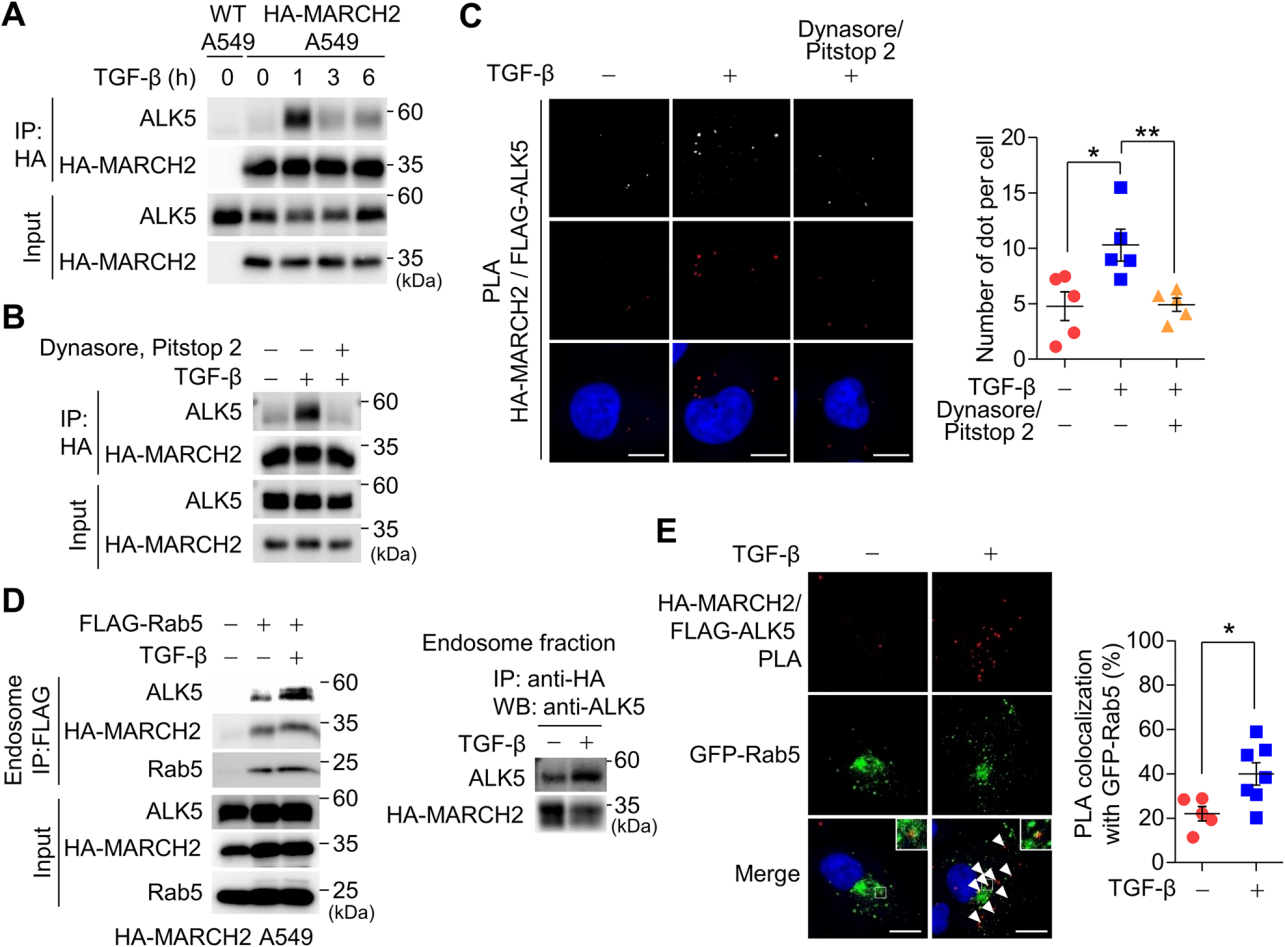
MARCH2 interacts with ALK5 at the endosomes in a TGF-β-dependent manner. **A** A co-IP assay was performed to examine the interaction between HA-MARCH2 and endogenous ALK5 following TGF-β treatment. A549 cells stably expressing HA-MARCH2 were treated with TGF-β for the indicated time periods. Proteins co-precipitated with HA-MARCH2 were analyzed by immunoblotting using an anti-ALK5 antibody. **B** A co-IP assay was conducted to investigate the interaction between HA-MARCH2 and endogenous ALK5 in the presence of the endocytosis inhibitors Dynasore (80 μM) and Pitstop 2 (30 μM). Cells were pre-treated with the inhibitors for 1 hour before TGF-β treatment for 3 h. Proteins co-precipitated with HA-MARCH2 were analyzed by immunoblotting using an anti-ALK5 antibody. **C** A proximity ligation assay (PLA) was performed to visualize the interaction between HA-MARCH2 and FLAG-ALK5 following TGF-β treatment. A549 cells stably expressing HA-MARCH2 and FLAG-ALK5 were pre-treated with Dynasore (80 μM) and Pitstop 2 (30 μM) for 1 h, followed by TGF-β treatment for 1 hour. Interaction was visualized using Texas Red fluorescence and analyzed using confocal microscopy. Fluorescence puncta were quantified using ImageJ software, and red fluorescence was converted to white for clarity. The number of puncta is presented in the graph on the right. Scale bar: 10 μm. **D** A co-IP assay was performed to examine the interaction between HA-MARCH2 and endogenous ALK5 at endosomes following TGF-β treatment. Endosomes were immunoprecipitated using FLAG-Rab5, and the isolated endosomes were subjected to a co-IP assay. Endogenous ALK5 co-precipitated with HA-MARCH2 was analyzed by immunoblotting with an anti-ALK5 antibody. **E** A PLA was performed in cells expressing GFP-Rab5 to visualize the interaction between HA-MARCH2 and FLAG-ALK5 within endosomes. A549 cells stably expressing GFP-Rab5 were treated with TGF-β for 1 h, followed by PLA analysis. The arrowheads indicate the colocalization of red puncta and green fluorescence from GFP-Rab5, and the inset shows a zoomed-in image highlighting the colocalization of PLA puncta with GFP-Rab5. Scale bar: 10 μm. Statistical analysis for (**C**, **E**) was performed using a two-tailed unpaired *t*-test and data are presented as the mean ± SD, where *p < 0.05, **p < 0.01.

Although the domain analyses of ALK5 and MARCH2 were performed using overexpressed proteins, we attempted to determine whether MARCH2 binding to ALK5 depends on TGF-β ligand stimulation. Our IP-mass spectrometry analysis identified ALK5 as a MARCH2 binding partner exclusively in TGF-β-treated samples (Fig. 2A), suggesting that ligand activation may be required for this interaction. To test this, we treated A549 cells stably expressing HA-MARCH2 with TGF-β for increasing time periods and performed co-IP assays. Endogenous ALK5 co-precipitated with HA-MARCH2 only in TGF-β-treated cells, with the interaction peaking at one hour and gradually declining thereafter (Fig. 3A). Since TGF-β receptors undergo endocytosis following TGF-β treatment and progress through distinct endosomal stages, we hypothesized that the MARCH2-ALK5 interaction occurs in early endosomes. To test this, we examined MARCH2-ALK5 interaction after blocking endocytosis using Dynasore and Pitstop 2, which inhibit dynamin- and clathrin-mediated endocytosis, respectively. Co-IP experiments revealed a significant reduction in the interaction when endocytosis was inhibited (Fig. 3B). To further confirm that MARCH2 and ALK5 interact within the endosomes, we conducted a proximity ligation assay (PLA) in A549 cells stably co-expressing HA-MARCH2 and FLAG-ALK5. Upon TGF-β treatment, we observed a significant increase in fluorescent puncta, indicating close proximity between MARCH2 and ALK5. However, this increase was suppressed to basal levels when cells were co-treated with Dynasore and Pitstop 2 (Fig. 3C). To validate these observations biochemically, we isolated FLAG-Rab5-containing endosomes via immunoprecipitation and assessed the interaction between MARCH2 and ALK5. Immunoblotting of endosomal precipitates revealed a robust MARCH2-ALK5 interaction following TGF-β treatment, which was markedly reduced in untreated cells (Fig. 3D). Furthermore, PLA puncta representing MARCH2-ALK5 interaction upon TGF-β treatment colocalized with GFP-Rab5 (Fig. 3E), confirming that these interactions occur in early endosomes. Collectively, these findings strongly suggest that MARCH2 predominantly interacts with ALK5 in the endosomal environment rather than at the plasma membrane, and that this interaction depends on endocytic trafficking in response to TGF-β stimulation.

### ALK5 is ubiquitinated by MARCH2

**Fig. 4:**
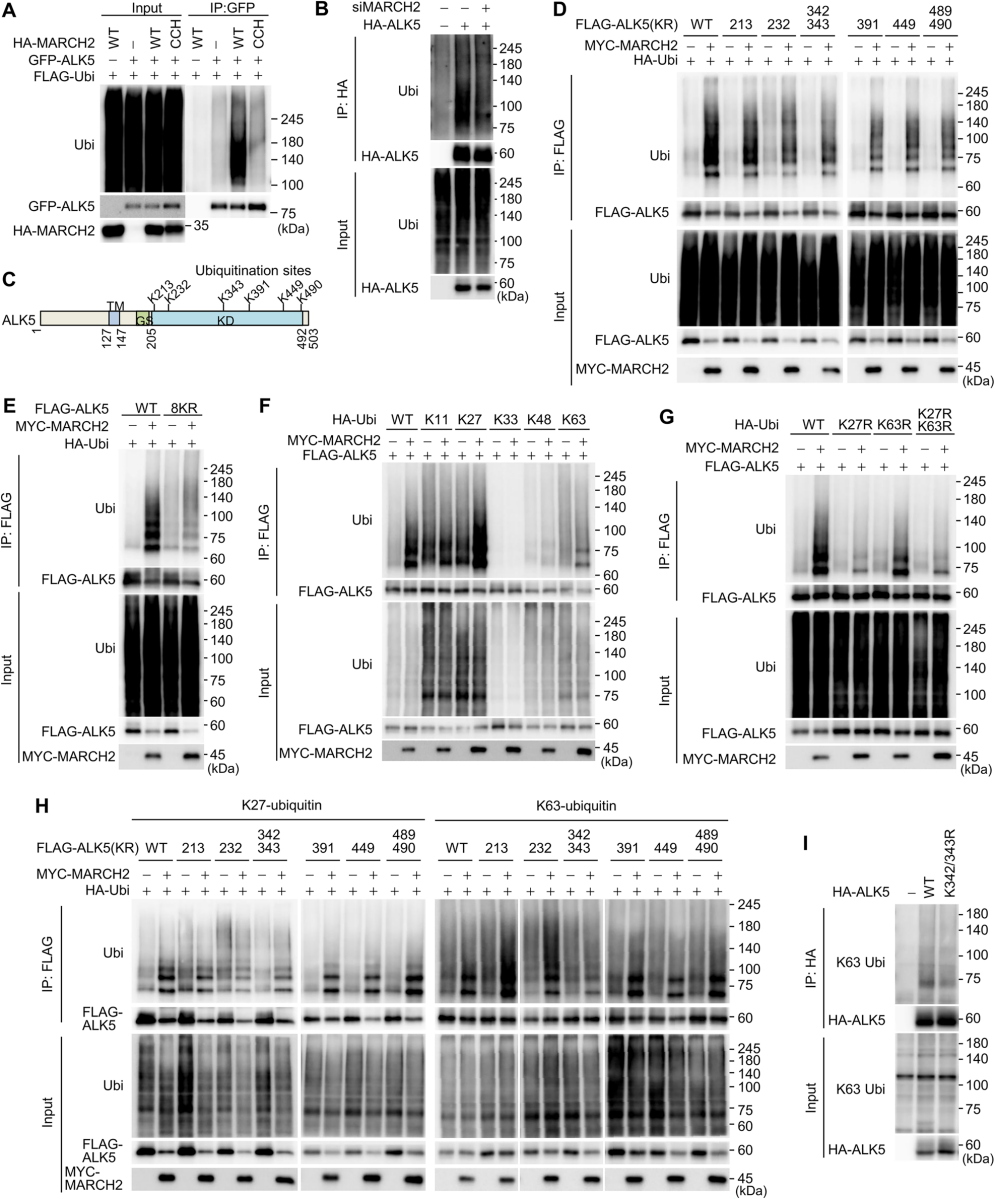
MARCH2 conjugates K27- and K63-linked ubiquitin chains onto ALK5. **A** A ubiquitination assay of ALK5 was performed in HEK293T cells co-transfected with HA-MARCH2, a catalytically inactive HA-MARCH2 mutant (CCH), FLAG-Ubiquitin, and GFP-ALK5. Ubiquitin-conjugated GFP-ALK5 was analyzed by immunoblotting with the indicated antibodies. **B** A ubiquitination assay of ALK5 was performed in MARCH2-depleted cells. HEK293 cells transfected with MARCH2-targeting siRNA were subsequently transfected with HA-ALK5 and treated with TGF-β for 1 h. Ubiquitin-conjugated HA-ALK5 was analyzed by immunoblotting with the indicated antibodies. **C** A schematic representation of ALK5 ubiquitination sites identified via mass spectrometry. **D** A ubiquitination assay of the ALK5 KR mutant was performed. HEK293 cells were co-transfected with the FLAG-ALK5 KR mutant, MYC-MARCH2, and HA-Ubiquitin. Ubiquitin-conjugated FLAG-ALK5 was analyzed by immunoblotting with the indicated antibodies. **E** HEK293T cells were co-transfected with MYC-MARCH2, HA-Ubiquitin, and either the FLAG-ALK5 or FLAG-ALK5 8KR mutant (K213R, K232R, K342/343R, K391R, K449R, and K489/490R). Ubiquitin-conjugated FLAG-ALK5 was analyzed by immunoblotting with the indicated antibodies. **F** A ubiquitination assay of ALK5 was performed using single-chain ubiquitin mutants. HEK293T cells were co-transfected with MYC-MARCH2, FLAG-ALK5, and HA-Ubiquitin mutants in which all lysines were substituted with arginines except for a single lysine residue (K11, K27, K33, K48, or K63). Ubiquitin-conjugated FLAG-ALK5 was analyzed by immunoblotting with the indicated antibodies. **G** A ubiquitination assay of ALK5 was performed using ubiquitin mutants defective in forming K27- or K63-linked chains. HEK293T cells were co-transfected with MYC-MARCH2, FLAG-ALK5, and HA-Ubiquitin mutants (K27R, K63R, or K27R/K63R). Ubiquitin-conjugated FLAG-ALK5 was analyzed by immunoblotting with the indicated antibodies. **H** A ubiquitination assay of ALK5 was performed using single-chain ubiquitin mutants. HEK293T cells were co-transfected with HA-Ubiquitin mutants (left panel: K27 only; right panel: K63 only), MYC-MARCH2, and individual FLAG-ALK5 KR mutants. Ubiquitin-conjugated FLAG-ALK5 was analyzed by immunoblotting with the indicated antibodies. **I** A ubiquitination assay of the ALK5 K342/343 R mutant was performed. HEK293T cells were transfected with either HA-ALK5 or the HA-ALK5 K342/343 R mutant. Ubiquitin-conjugated HA-ALK5 was analyzed by immunoblotting with an anti-K63 ubiquitin antibody.

To investigate whether MARCH2 ubiquitinates ALK5 and to identify specific ubiquitination sites, we transfected HEK293T cells with either HA-MARCH2, GFP-ALK5, or both constructs. Immunoblotting of immunoprecipitated GFP-ALK5 with an anti-ubiquitin antibody showed that ALK5 was ubiquitinated by MARCH2 (Fig. 4A). This ALK5 ubiquitination was induced by wild-type MARCH2 but not by a catalytically inactive mutant (CCH), indicating that MARCH2 E3 ligase activity is responsible for ALK5 ubiquitination (Fig. 4A). To further validate these findings, we examined ALK5 ubiquitination in MARCH2-depleted cells. The ubiquitination levels of overexpressed HA-ALK5 decreased upon MARCH2 depletion (Fig. 4B), reinforcing the role of MARCH2 in ALK5 ubiquitination. To identify the specific ALK5 ubiquitination sites targeted by MARCH2, we co-expressed FLAG-ALK5 with MARCH2 and analyzed immunoprecipitated FLAG-ALK5 by mass spectrometry. This analysis identified six lysine residues (K213, K232, K343, K391, K449, and K490) as ubiquitination sites (Fig. 4C and Fig. S2). Further examination using individual ALK5 K-to-R mutants revealed that none of the single mutations affected ALK5 ubiquitination, suggesting that multiple lysine residues are ubiquitinated simultaneously (Fig. 4D). Given the potential redundancy among adjacent lysine residues, we generated additional mutations in which adjacent lysines were mutated together (e.g., K342/343 R and K489/490 R). Notably, ubiquitination was completely abolished when all eight lysines were substituted with arginine (8KR mutant), indicating that MARCH2 ubiquitinates ALK5 at multiple sites concurrently (Fig. 4E). Since the functional impact of ubiquitination is often linked to the type of ubiquitin chains formed, we examined the specific ubiquitin linkages catalyzed by MARCH2. When FLAG-ALK5 was co-expressed with ubiquitin mutants, with or without MYC-MARCH2, immunoblotting revealed that MARCH2 predominantly induced K27-linked ubiquitin chains and, to a lesser extent, K63-linked chains on ALK5. In contrast, K11, K33, and K48 ubiquitin conjugates were not detected (Fig. 4F). To confirm these findings, we co-expressed ALK5 with K27R or K63R ubiquitin mutants. ALK5 ubiquitination was markedly reduced when the K27R ubiquitin mutant was present, with a slight reduction observed for the K63R mutant (Fig. 4G). These results suggest that MARCH2 primarily catalyzes K27-linked ubiquitination of ALK5, as well as K63-linked ubiquitination to a lesser extent. To further identify the specific ALK5 lysine residues targeted for K27- and K63-linked ubiquitination, we assessed the ubiquitination patterns of individual ALK5 K-to-R mutants using ubiquitin constructs that exclusively form K27- or K63-linked chains. Immunoblotting revealed that K27-linked ubiquitination was unaffected in all single mutants except K232R, suggesting that K232 is a major site for K27-linked ubiquitination (Fig. 4H, left panel). In contrast, K63-linked ubiquitination was abolished in the ALK5 K342/343 R mutant, indicating that these residues are the primary sites for K63-linked ubiquitination (Fig. 4H, right panel). To validate these findings, we performed immunoblotting using a K63-specific ubiquitin antibody. A significant reduction in K63-linked ubiquitination was observed in the ALK5 K342/343 R mutant (Fig. 4I), further confirming that K342/343 are critical for K63-linked ubiquitination. Finally, to confirm the specific lysine residues involved in K27- and K63-linked ubiquitination, we examined ubiquitination in lysine revertant mutants of ALK5, in which individual lysine residues were restored in the 8KR background. Immunoblotting revealed that ubiquitination was primarily restored in the K232 and K342/343 revertants upon expression of wild-type ubiquitin and MARCH2 (Fig. S3A). Further analysis confirmed that K232 is the primary site for K27-linked ubiquitination (Fig. S3B, left panel). Additionally, K63-linked ubiquitination was restored predominantly in the K342/343 revertant, with a minor contribution from K232 (Fig. S3B, right panel), confirming that these residues are key sites for K63-linked ubiquitination. Taken together, these results demonstrate that MARCH2 mediates ALK5 ubiquitination through K27-linked ubiquitin chains at K232, while specifically targeting K342/343 and K232 for K63-linked ubiquitination.

### MARCH2-mediated ALK5 ubiquitination affects ALK5 catalytic activity

**Fig. 5:**
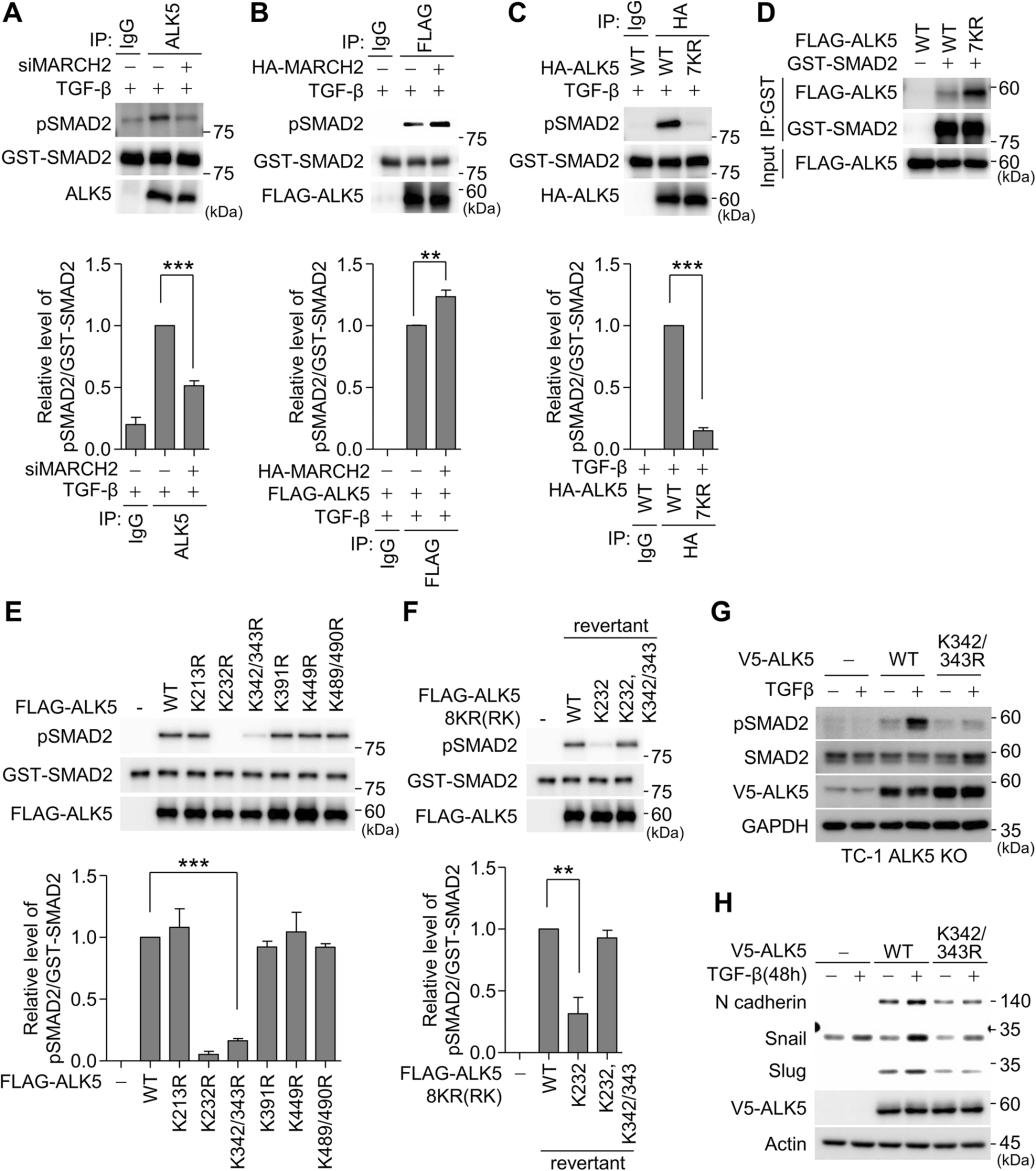
MARCH2-mediated ALK5 ubiquitination regulates ALK5 catalytic activity. **A** An immunoprecipitation (IP) kinase assay was performed in A549 cells transfected with the indicated siRNA and treated with TGF-β for 1 h. Endogenous ALK5 was immunoprecipitated and incubated with purified GST-SMAD2. Catalytic activity was determined by immunoblotting with an anti-phospho-SMAD2 antibody. **B** FLAG-ALK5 isolated from HEK293 cells transiently expressing FLAG-ALK5, with or without HA-MARCH2, was subjected to an IP kinase assay. Cells were treated with TGF-β for 1 h. The immunoprecipitated FLAG-ALK5 was incubated with purified GST-SMAD2, and catalytic activity was analyzed by immunoblotting with an anti-phospho-SMAD2 antibody. **C** HA-ALK5 or the HA-ALK5 7KR mutant, isolated from A549 cells, was subjected to an IP kinase assay. Cells were treated with TGF-β for 1 hour. The immunoprecipitated HA-ALK5 or HA-ALK5 7KR was incubated with purified GST-SMAD2, and catalytic activity was analyzed by immunoblotting with an anti-phospho-SMAD2 antibody. **D** An in vitro binding assay was performed to assess the interaction between FLAG-ALK5 or FLAG-ALK5 7KR and GST-SMAD2. Cell lysates from HEK293 cells expressing FLAG-ALK5 or FLAG-ALK5 7KR were incubated with GST-SMAD2, and protein interactions were analyzed by immunoblotting with the indicated antibodies. **E** FLAG-ALK5 or FLAG-ALK5 single KR mutants, isolated from HEK293 cells, were subjected to an IP kinase assay. The immunoprecipitated FLAG-ALK5 or mutants were incubated with purified GST-SMAD2, and catalytic activity was analyzed by immunoblotting with an anti-phospho-SMAD2 antibody. **F** FLAG-ALK5 or FLAG-ALK5 revertants (K232 and K232/K342/343), isolated from HEK293 cells, were subjected to an IP kinase assay. The immunoprecipitated FLAG-ALK5 or mutants were incubated with purified GST-SMAD2, and catalytic activity was analyzed by immunoblotting with an anti-phospho-SMAD2 antibody. Quantification of relative pSMAD2 levels, normalized to GST-SMAD2 levels, are presented in the graphs (**A**–**C**, **E**, **F**). **G** TGF-β signaling activation was examined in TC-1 ALK5 knockout (KO) cells and reconstituted TC-1 ALK5 KO cells stably expressing either V5-tagged ALK5 WT or the V5-ALK5 K342/343 R mutant after TGF-β treatment for 1 h. Activation of TGF-β signaling was analyzed by immunoblotting with the indicated antibodies. **H** Expression of EMT markers was analyzed in TC-1 ALK5 KO cells and reconstituted TC-1 ALK5 KO cells stably expressing either V5-ALK5 WT or the V5-ALK5 K342/343 R mutant after 48 h of TGF-β treatment. EMT marker expression was assessed by immunoblotting with the indicated antibodies.

To investigate how MARCH2-mediated ubiquitination influences ALK5 function, we first examined whether MARCH2 promotes K48-linked ubiquitination, which typically signals protein degradation. Immunoblotting confirmed that MARCH2 does not mediate K48-linked ubiquitination of ALK5 (Fig. 4F). Consistently, ALK5 stability remained unchanged following MARCH2 depletion (Fig. S4A). Next, we evaluated whether MARCH2 affects ALK5 internalization, a process that reduces ALK5 levels from the plasma membrane upon TGF-β binding. Immunoblotting of biotinylated plasma membrane fractions revealed that ALK5 internalization was not altered by MARCH2 depletion (Fig. S4B). These findings suggest that MARCH2-mediated ubiquitination does not regulate ALK5 stability or cellular localization during TGF-β signaling. Since SMAD2/3 phosphorylation was reduced in MARCH2-depleted cells (Fig. 1A), we hypothesized that ALK5 ubiquitination is required for SMAD2/3 activation. To determine whether the reduction in SMAD2/3 phosphorylation in MARCH2-depleted cells was due to impaired SMAD2/3 recruitment, we performed a proximity ligation assay (PLA) to assess ALK5-SMAD2/3 interaction in MARCH2-depleted cells. The PLA revealed no significant changes in ALK5-SMAD2/3 interaction upon TGF-β treatment (Fig. S4C), suggesting that MARCH2-mediated ALK5 ubiquitination does not affect SMAD2/3 binding. We next examined whether ALK5 ubiquitination influences its catalytic activity. ALK5 was immunoprecipitated from mock- or MARCH2-depleted cells and subjected to kinase assays using purified GST-SMAD2 as a substrate. Immunoblotting with phospho-specific anti-SMAD2 antibodies demonstrated that ALK5 from mock-depleted cells efficiently phosphorylated GST-SMAD2, whereas ALK5 from MARCH2-depleted cells showed significantly reduced phosphorylation activity (Fig. 5A). Conversely, SMAD2 phosphorylation was enhanced by ALK5 from cells overexpressing MARCH2 (Fig. 5B). To determine whether ubiquitination is required for ALK5 catalytic activity, we examined the activity of the ALK5 7KR mutant in which seven lysine residues except K232 (required for ATP binding) were mutated to arginine. The 7KR mutant exhibited a complete loss of kinase activity toward GST-SMAD2 (Fig. 5C), and this reduction was not due to impaired SMAD2 binding (Fig. 5D). These results demonstrate that MARCH2-mediated ubiquitination enhances ALK5 catalytic activity, thereby facilitating SMAD2 phosphorylation. To further analyze the role of individual ubiquitination sites, we evaluated the kinase activity of ALK5 K-to-R mutants. All mutants efficiently phosphorylated GST-SMAD2 except for K232R and K342/343 R. Since K232R is catalytically inactive due to defective ATP binding, the reduced catalytic activity of K342/343 R specifically reflects the absence of MARCH2-mediated ubiquitination at K342/343 (Fig. 5E). Furthermore, the reduced catalytic activity observed in the 7KR mutant (K232 revertant) was fully restored when K342/343 were reintroduced (Fig. 5F), demonstrating that ubiquitination at K342/343 is sufficient to enhance ALK5 catalytic activity. To confirm the effect of K342/343 mutation on the ALK5 catalytic activity in cells, we generated cell lines expressing ALK5 K342/343 R mutant in the ALK5 KO background (Fig. S5A). Immunoblotting revealed that SMAD2 phosphorylation was abolished in cells expressing the ALK5 K342/343 R mutant, but remained robust in cells expressing ALK5 WT (Fig. 5G). Consistently, expression of TGF-β target genes, such as N-cadherin, Snail, and Slug, were not induced in cells expressing the ALK5 K342/343 R mutant (Fig. 5H). These results establish K342/343 as ubiquitination sites critical for SMAD2 phosphorylation. Taken together, these findings demonstrate that MARCH2-mediated ubiquitination of ALK5 at K342/343 is crucial for its catalytic activity, enabling efficient SMAD2 phosphorylation and TGF-β signaling.

### Ubiquitination of ALK5 by MARCH2 regulates cell migration, invasion, and metastasis

**Fig. 6:**
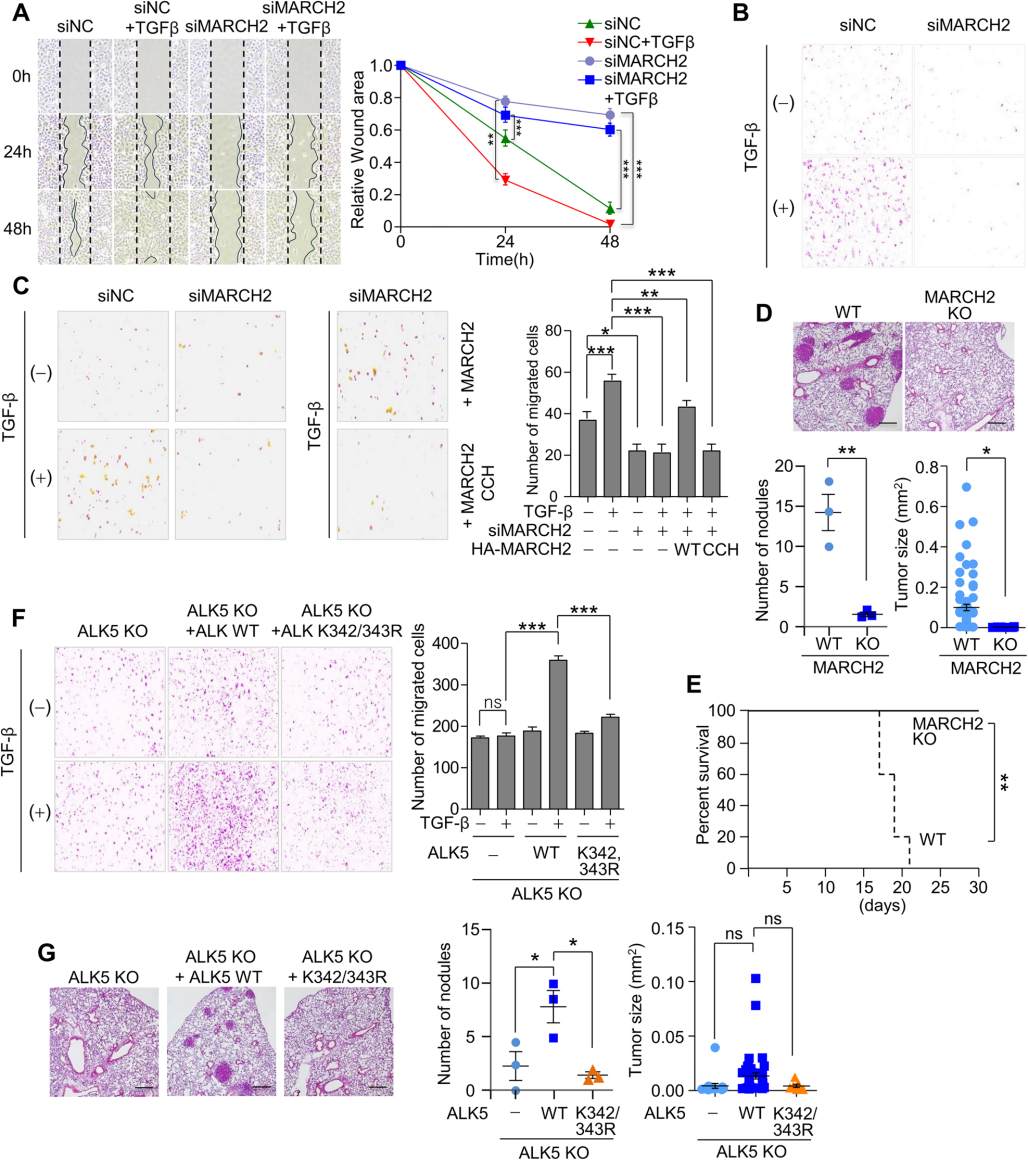
MARCH2-mediated ALK5 ubiquitination regulates cell migration, invasion, and metastasis. **A** A wound healing assay was performed using A549 cells depleted of MARCH2. A549 cells were transfected with siRNA targeting MARCH2, and a single-line scratch wound was created 24 h post-transfection. Cells were then incubated with or without TGF-β. Wound closure was monitored at designated time points, and the wound area was quantified using ImageJ software. Each experiment was repeated three times, and quantification results are presented in the graph on the right. **B** A transwell invasion assay was performed using A549 cells depleted of MARCH2. A549 cells (0.5 × 10^5^) or MARCH2-depleted A549 cells were seeded in the upper chamber with low-serum medium, while TGF-β was added to the lower chamber containing high-serum medium. Migrated cells were stained, counted, and quantified. Each experiment was repeated three times. **C** A transwell invasion assay was performed using HCT116 cells expressing either MARCH2 WT or a catalytically inactive MARCH2 mutant (CCH) under TGF-β treatment. Results were quantified and presented in the graph on the right. **D** A tail-vein injection assay was performed to assess the metastatic potential of MARCH2 KO TC-1 cells. Lung tumors in mice were visualized by H&E staining, and tumor nodule number and size were quantified. Scale bar: 250 μm. Quantified lung nodule number (*n* = 3) and tumor size data are presented in the graph. **E** To induce lung metastasis, WT or MARCH2 KO TC-1 cells were injected into the tail vein. The survival rates were determined and are displayed in the graph (*n* = 5). **F** A transwell invasion assay was performed using A549 ALK5 KO cells expressing either ALK5 WT or the K342/343 R mutant under TGF-β treatment. Invaded cells were stained, counted, and quantified. Each experiment was repeated three times. **G** A tail vein injection assay was performed to evaluate the metastatic potential of TC-1 cells expressing the ALK5 K342/343 R mutant. Lung tumors were visualized by H&E staining, and tumor nodule number and size were quantified. Scale bar: 250 μm. Quantified lung nodule number (*n* = 3) and tumor size data are presented in the graph. Statistical analysis for (**A**, **C**–**G**) was performed using a two-tailed unpaired *t*-test and data are presented as the mean ± SD, where *p < 0.05, **p < 0.01, ***p < 0.001, ns (not significant).

To investigate the effects of MARCH2 depletion on the cellular response to TGF-β signaling, we examined TGF-β-induced cell migration and invasion. Since SMAD2 phosphorylation was impaired in MARCH2-depleted cells (Fig. 1A), we assessed wound closure using a scratch assay. In mock-depleted cells, TGF-β treatment significantly enhanced wound closure, whereas in MARCH2-depleted cells, wound closure was minimal even in the presence of TGF-β (Fig. 6A). To further evaluate the impact of MARCH2 depletion, we conducted a transwell invasion assay. TGF-β treatment significantly increased cell invasion in mock-depleted cells, while this invasion was markedly impaired in MARCH2-depleted cells (Fig. 6B). Notably, reintroduction of an siRNA-resistant MARCH2 construct rescued cell invasion, whereas a catalytically inactive MARCH2 mutant did not (Fig. 6C). These findings indicate that the E3 ligase activity of MARCH2 is essential for TGF-β-induced cell migration and invasion, likely through its ubiquitination function. To validate these findings in vivo, we utilized a mouse model. In a subcutaneous tumor formation assay, MARCH2-knockout (KO) TC-1 cells (Fig. S5B) showed a marked reduction in primary tumor formation compared to wild-type (WT) TC-1 cells (Fig. S5C, S5D). Furthermore, upon assessment of the metastatic potential of WT and MARCH2 KO cells using a tail-vein injection assay, WT cells efficiently metastasized to the lungs, whereas MARCH2 KO cells exhibited dramatically reduced metastatic potential (Fig. 6D). These results confirm that MARCH2 is required for tumor metastasis and, consequently, loss of MARCH2 significantly increased the survival rate of the mice (Fig. 6E). We next investigated whether ALK5 ubiquitination plays a role in these processes. Using an ALK5 K342/343 R mutant resistant to MARCH2-mediated K63-linked ubiquitination, we conducted a transwell invasion assay. ALK5 KO cells reconstituted with wild-type ALK5 exhibited robust TGF-β-induced invasion, while cells expressing the ALK5 K342/343 R mutant failed to invade in response to TGF-β (Fig. 6F). Similarly, in a tail-vein injection assay, ALK5 KO cells expressing wild-type ALK5 regained metastatic potential, whereas cells expressing the ALK5 K342/343 R mutant did not (Fig. 6G). Taken together, these findings demonstrate that MARCH2-mediated ubiquitination of ALK5 is essential for TGF-β-driven cell migration, invasion, and metastasis. Specifically, ubiquitination at K342/343 is a critical regulatory mechanism in TGF-β signaling-dependent tumor metastasis.

### Expression of MARCH2 correlates with TGF-β target gene expression in various tumor types

**Fig. 7:**
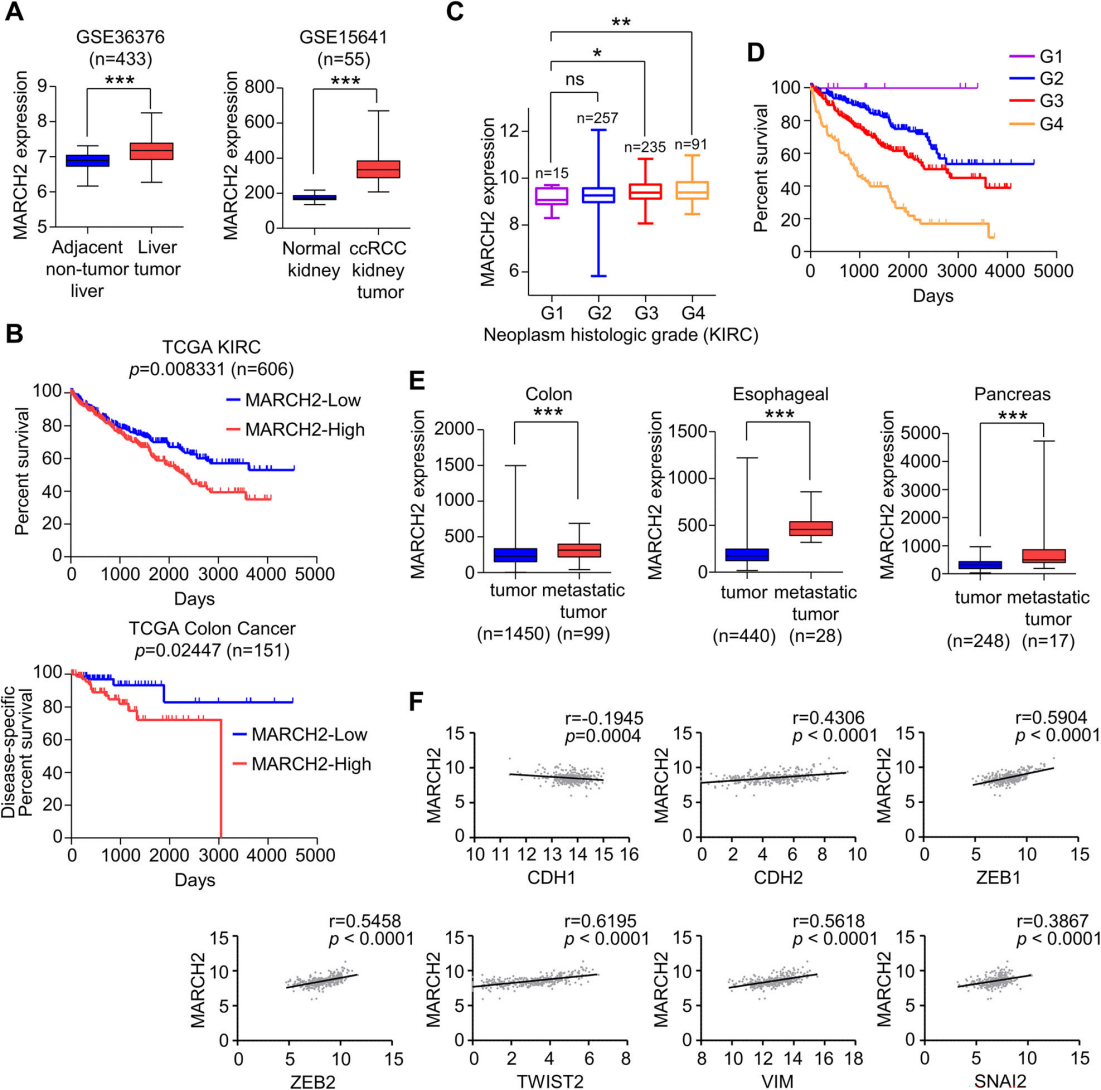
MARCH2 expression is upregulated in tumors and correlates with TGF-β target gene expression. **A** MARCH2 expression levels in kidney clear cell carcinoma and hepatocellular carcinoma tissues were compared with those in normal tissues using public GEO datasets (GSE36376, *n* = 433; GSE15641, *n* = 55). **B** Kaplan-Meier analysis of TCGA data revealed a correlation between MARCH2 expression and overall survival rates in human kidney clear cell carcinoma (TCGA KIRC, *n* = 606). Additionally, disease-specific survival rates were compared between the 1st and 4th quartiles of colon cancer patients (TCGA colon cancer, *n* = 151). **C** MARCH2 expression levels were analyzed according to tumor grade using TCGA kidney renal clear cell carcinoma (KIRC) data. **D** Kaplan-Meier analysis of TCGA data showed a correlation between MARCH2 expression and overall survival rates across different histologic grades of human kidney clear cell carcinoma (KIRC). **E** MARCH2 expression levels in metastatic tumor tissues (colon, esophagus, and pancreas) were compared with those in non-metastatic tumor tissues using public TNM plot datasets. Statistical analysis for (**A**, **C**, **E**) was performed using a two-tailed unpaired *t*-test and data are presented as the mean ± SD, where *p < 0.05, **p < 0.01, ***p < 0.001, ns (not significant). **F** The correlation between MARCH2 mRNA levels and TGF-β target genes in human colon cancer patients was analyzed using TCGA datasets. The Pearson correlation coefficient (r) and corresponding *p* values are indicated.

To further investigate the role of MARCH2 in human cancers, we analyzed gene expression data from Gene Expression Omnibus Series (GEO). This analysis revealed that MARCH2 expression levels were significantly elevated in liver and kidney tumors compared to adjacent normal tissues (Fig. 7A). Additionally, survival curve analysis using data from The Cancer Genome Atlas (TCGA) demonstrated that high MARCH2 expression was associated with reduced survival rates in patients with clear cell renal cell carcinoma and colon cancers (Fig. 7B). Further analysis showed that MARCH2 expression progressively increased with higher histologic grades of neoplasms (Fig. 7C) and exhibited an inverse correlation with survival rates across different histologic grades of clear cell renal cell carcinoma (Fig. 7D). Notably, data from TNM plot analysis [34] indicated that MARCH2 expression levels were higher in metastatic tumors compared to non-metastatic tumors in colon, esophageal, and pancreatic cancers (Fig. 7E). Consistent with the hypothesis that MARCH2 enhances ALK5 activity and promotes TGF-β signaling, TCGA data analysis revealed a strong positive correlation between MARCH2 expression and the expression of TGF-β target genes in colon (Fig. 7F) and stomach cancers (Fig. S6). Taken together, these findings suggest that MARCH2 functions as an upstream regulator of TGF-β signaling by modulating the ubiquitination-mediated catalytic activity of the TGF-β type I receptor, thereby driving the expression of TGF-β target genes in various human cancers.

## Discussion

TGF-β signaling is initiated when the constitutively active TGF-β type II receptor phosphorylates the TGF-β type I receptor (ALK5) upon ligand binding [30, 35]. The resulting receptor complex undergoes endocytosis via both clathrin- and caveolae-mediated pathways, which distinctly regulate TGF-β signal propagation and attenuation, respectively [36, 37]. Although TGF-β receptor internalization is not strictly required for SMAD2/3 phosphorylation and downstream signaling [38], it significantly influences signal duration and intensity by enhancing receptor stability and facilitating the recruitment of adaptor proteins, such as Smad anchor for receptor activation (SARA) [39–41]. In this study, we demonstrate that MARCH2 interacts with ALK5 in the endosomes in a TGF-β-dependent manner (Fig. 3). The disruption of the MARCH2-ALK5 interaction in cells treated with endocytosis inhibitors suggests that the endosomal environment is crucial for their interaction (Fig. 3B, C). During clathrin-mediated endocytosis, the AP-2 complex, a heterotetramer composed of the α, β2, μ2, and σ2 subunits, bridges cargo proteins to clathrin and accessory proteins [42]. Mass spectrometry analysis identified the α and β subunits of the AP-2 complex, along with EEA1, as TGF-β-dependent MARCH2-interacting proteins (Table S1 and Fig. S7A). This suggests that MARCH2 can be enriched in endosomes via its association with AP-2 during clathrin-mediated endocytosis and stabilized through interaction with the endosomal adaptor protein EEA1. Domain analysis indicated that the transmembrane domains (TMs) of MARCH2 and ALK5 play a key role in their interaction (Fig. 2F, J). Heptad repeats within these TMs promote helical coiled-coil interactions, where hydrophobic residues at the a and d positions facilitate “knobs-into-holes” packing [43, 44]. Helical wheel diagrams of the MARCH2 and TGF-β receptor TMs revealed that hydrophobic residues occupying the a and d positions of the heptad repeats in MARCH2 TM2 and ALK5 TM align favorably, whereas such a match does not occur between MARCH2 TM1 and TGFBR2 TM (Fig. S7B). This suggests that TM-TM interactions contribute to the association of full-length MARCH2 and ALK5. Additionally, endosomal membrane curvature and lipid composition may enhance this interaction through the TM domains [44]. Endosomes formed via clathrin-mediated endocytosis are rich in phosphatidylinositol 3-phosphate (PI3P), a lipid critical for recruiting PI3P-binding proteins with Phox Homology (PX) domains or FYVE motifs, such as EEA1 and SARA [45, 46]. The high PI3P concentration creates a specialized lipid microenvironment that increases membrane curvature and facilitates the recruitment of adaptor proteins [47]. Collectively, these factors likely establish an optimal setting for MARCH2 and ALK5 to interact via their TM domains within curved or tensioned endosomal membranes. Given that MARCH2-mediated ubiquitination of ALK5 in the endosomes is essential for its full catalytic activation, the endosomes serve as a critical platform for ALK5 enzymatic activation. Furthermore, this environment supports the efficient recruitment of SMAD2/3 through the endosome-associated adaptor protein SARA, reinforcing the role of endosomal trafficking in fine-tuning TGF-β signaling.

Protein kinases are regulated at multiple levels, with ubiquitination influencing abundance and phosphorylation modulating catalytic activity [48]. ALK5 is activated through phosphorylation at multiple sites within its GS domain by the TGF-β type II receptor. Its abundance is negatively regulated by a Smad7-mediated feedback loop, where Smurf1/2 ubiquitinates ALK5 for proteasomal degradation [49]. In this study, we provide evidence that the catalytic activity of ALK5 is regulated by MARCH2-mediated K63-linked ubiquitination at K342/343 (Fig. 5E, F, and Fig. S3B). Previous studies have shown that TRAF6 activates protein kinases through K63-linked polyubiquitination, where ubiquitination of IKKβ at K147 and TAK1 at K158 is crucial for their catalytic activity and substrate phosphorylation [50, 51]. Amino acid alignment of IKKβ, TAK1, BRAF, MLK3, ZAP-70, and ALK5 reveals that the lysine residues subjected to K63-linked ubiquitination are located between the HRD and DFG motifs within their catalytic loops (Fig. S7C). Furthermore, K147 of IKKβ, K158 of TAK1, and K578 of BRAF occupy equivalent positions two amino acids downstream from the HRD motif [52], while K342/343 of ALK5 are situated seven amino acids away within the same region. This suggests that K63-linked ubiquitination within the catalytic loop is a conserved mechanism for regulating protein kinase activity. Quantitative mass spectrometry analyses indicate that non-degradative ubiquitination frequently occurs within structured regions of the kinase domain, which are resistant to proteasomal degradation. Specifically, ubiquitination is enriched in the catalytic loop near the DFG motif, N-terminal to the activation loop, and in the glycine-rich loop [53, 54]. Molecular simulations on ZAP-70 illustrate that ubiquitination at different lysine residues can have opposing effects. Ubiquitination at K377 disrupts activity, whereas K476 ubiquitination enhances catalytic activity [54]. Notably, K476 ubiquitination in ZAP-70, located three amino acids upstream from the DFG motif, aligns with the K63-linked ubiquitination site of MLK3 (Fig. S7C), which is essential for its catalytic activity [55]. Taken together, these findings suggest that K63-linked ubiquitination at lysine residues located between the HRD and DFG motifs within the catalytic loop stabilizes the active conformation of kinases, thereby modulating their catalytic activity. Given that many protein kinases, including Lck, MAPK8, MAPK9, MAPK10, and CDK5, are ubiquitinated in this region [54], further studies are warranted to validate the hypothesis that K63-linked ubiquitination within the catalytic loop represents a conserved mechanism for regulating protein kinase activity.

Given our findings regarding MARCH2-mediated regulation of the TGF-β pathway and its impact on tumor metastasis, it is crucial to consider how this E3 ligase contributes to the broader landscape of target protein regulation through ubiquitination. Similar to other MARCH family proteins such as MARCH1, MARCH5, MARCH8, and MARCH9, MARCH2 plays context-dependent roles in cancer progression depending on the tumor type and cellular environment [17]. Our current findings indicate that MARCH2 promotes TGF-β signaling and metastasis. This aligns with its observed oncogenic roles in knock-out mouse models, where the absence of MARCH2 led to inhibited tumor growth and proliferation, while promoting autophagy, apoptosis, and G2/M cell cycle arrest [56]. Furthermore, studies on colon cancer models have also demonstrated the oncogenic impact of MARCH2 overexpression [18]. Conversely, other research has shown that MARCH2 can target Snail for ubiquitination and proteasomal degradation, and that PTK6 regulates MARCH2 levels in the context of Snail-mediated EMT in triple-negative breast cancer cells [20]. Therefore, future research is warranted to understand the dual roles of MARCH2 and to uncover the mechanistic basis for context-dependent functions of MARCH2 in cancer progression and metastatic potential.

## Methods

### Cell culture

HEK293T, HEK293, A549, HCT116, and TC-1 were purchased from the American Type Culture Collection (ATCC). HEK293T was cultured in Dulbecco’s Modified Eagle Medium (DMEM), supplemented with 10% fetal bovine serum (FBS) (Gibco) and 1% antibiotic-antimycotic. A549, HCT116, and TC-1 cells were cultured in RPMI 1640 (Gibco), supplemented with 10% FBS (Gibco) and 1% antibiotic-antimycotic. All cells were maintained at 37 °C in a humidified chamber with 5% CO_2_.

### Generation of knockout cell lines using CRISPR-Cas9 and establishment of stable cell lines

MARCH2 or ALK5 was knocked out using the CRISPR/Cas9 method. Oligonucleotides encoding single-guide RNAs (sgRNAs) were cloned into the LentiCRISPR v2 backbone. For lentivirus production, HEK293T cells were transfected with LentiCRISPR v2, Gag-pol, and VSV-G for 48 h. The lentivirus was then collected and applied to target cells for 24 h. Infected cells were selected using puromycin. Single colonies were isolated and screened, and gene knockout was confirmed by both immunoblotting and sequencing. Wild-type ALK5 and ALK5 K-to-R mutants were cloned into the pLJC5 or pLenti6 plasmid. Stably expressing cell lines were generated by recombinant lentiviral infection, followed by selection using puromycin or blasticidin.

### Plasmid construction

Full-length human ALK5 cDNA was obtained from Addgene (plasmid #80876). Full-length human MARCH2 cDNA was obtained from the Korea Human Gene Bank (Clone ID: hMU005086). Full-length ALK5, a deletion mutant of ALK5, MARCH2, and a deletion mutant of MARCH2 were amplified by PCR and cloned into the *ClaI* and *EcoRV* sites of the pCS5 + 3x HA or pCS5 + 3X FLAG vector (kindly provided by Dr. Jaewhan Song). For BiFC analysis, MARCH2 was cloned into the *EcoRI* and *XhoI* sites of pBiFC-VC155 (Addgene Plasmid #22011), and ALK5 was cloned into the *NotI* and *ClaI* sites of pBiFC-VN173 (Addgene Plasmid #22010). ALK5 KR mutants (K213R, K232R, K342/343 R, K391R, K449R, K489/490 R) and the MARCH2 catalytically inactive mutant (C64S, C67S, and H90Q) were generated using the Muta-Direct^TM^ Site-Directed Mutagenesis Kit (iNtRON, #15071). All PCR-generated constructs were verified by sequencing.

### Antibodies

Anti-MARCH2 (A13497) was purchased from Boster Bio. Anti-ALK5 (TGFβR1) (ab51871) and anti-ubiquitin (linkage-specific K63) (EPR8590-448) were purchased from Abcam. Anti-β-actin (4967), anti-N-cadherin (4061), anti-E-cadherin (3195), anti-Vimentin (5741), anti-pSMAD2 (3108), anti-SMAD2 (5339), anti-pSMAD3 (9520), anti-SMAD3 (9523), anti-FLAG (14793), anti-Snail (3895), and anti-Slug (9585) were purchased from Cell Signaling Technology. HA-horseradish peroxidase (HRP) (11-814-150-001) and c-Myc-HRP (12-013-819-001) were purchased from Roche. Anti-HA (sc-7392) and anti-c-Myc (sc-764) were purchased from Santa Cruz Biotechnology. Anti-GAPDH (A300-639A) was purchased from Bethyl Laboratories. Anti-α-Tubulin (05-829) and anti-ubiquitin (04-263) were purchased from Merck Millipore.

### Cell lysis and immunoblotting

Cells were lysed in RIPA lysis buffer (0.1% SDS, 0.1% sodium deoxycholate (SDC), 1% NP-40, 20 mM Tris–HCl, pH 7.6, 150 mM NaCl, 2 mM EDTA, 10 mM NaF, 1 mM Na_3_VO_4_, and a protease inhibitor cocktail). The lysates were centrifuged at 16,000 × g at 4 °C for 15 min. The supernatants were transferred to a new tube, boiled with sample buffer at 100 °C for 10 minutes, and subjected to SDS-PAGE. Proteins were transferred onto a PVDF membrane, which was blocked with either 4% skim milk or 4% BSA. The membrane was incubated with the indicated primary antibodies, followed by washing with TBST. The membrane was then incubated with secondary antibodies for 1 h and washed again with TBST. Protein signals were detected using ECL solution (Thermo Scientific, #34580).

### Immunoprecipitation

Cell lysates were prepared in lysis buffer (1% NP-40, 20 mM Tris–HCl, pH 7.6, 150 mM NaCl, 2 mM EDTA, 10 mM NaF, 1 mM Na_3_VO_4_, and a protease inhibitor cocktail) and incubated with the indicated magnetic beads under rotation overnight at 4 °C. The bead-protein complex was washed five times with lysis buffer and boiled with 2.5× sample buffer at 100 °C for 10 minutes. Immunoblot analysis was performed using the indicated antibodies.

### Immunofluorescence assay

Cells were cultured on coverslips and transfected with siRNA. After 48 h, cells were fixed with 3% paraformaldehyde for 10 minutes and permeabilized with 0.1% Triton X-100 for 5 min. Following permeabilization, 2% BSA was used for blocking, and cells were incubated with the indicated primary antibody in 2% BSA at room temperature for 2 h. Afterward, cells were incubated with fluorescence-conjugated secondary antibodies in PBS containing 2% BSA, along with DAPI for nuclear staining, for 20 min. A Zeiss LSM 700 confocal microscope and ZEN software were used to analyze immunofluorescence.

### BiFC assay

HeLa cells were transfected with plasmids encoding MARCH2-VC155 and ALK5-VN173. After 24 h, the cells were fixed with 3% paraformaldehyde for 10 min and permeabilized with 0.1% Triton X-100 for 5 minutes. Nuclei were stained with DAPI. Fluorescence was analyzed using a Zeiss LSM 700 confocal microscope and ZEN software.

### Ubiquitination assay

Cell lysates were prepared in RIPA lysis buffer (1% SDS, 0.1% SDC, 1% NP-40, 20 mM Tris–HCl, pH 7.6, 150 mM NaCl, 2 mM EDTA, 10 mM NaF, 1 mM Na_3_VO_4_, and a protease inhibitor cocktail) and boiled at 100 °C for 10 min. Lysates were sonicated until the solution was clear. The samples were then centrifuged at 16,000 × g at 4 °C for 15 min, and the supernatants were transferred to a new tube. The supernatant was diluted 10-fold with RIPA lysis buffer to reduce the SDS concentration to 0.1%. The supernatant was incubated with the indicated magnetic beads under rotation overnight at 4 °C. The next day, the bead-protein complex was washed five times with lysis buffer and boiled with 2.5× sample buffer at 100 °C for 10 min. Immunoblot analysis was performed using the indicated antibodies.

### Proximity ligation assay (PLA)

Cells were grown on coverslips, fixed with 3% paraformaldehyde for 10 min, and permeabilized with 0.1% Triton X-100 for 5 min. The PLA assay was performed using the Duolink® PLA Kit (DUO92101) following the manufacturer’s protocol. The slides were incubated with Duolink® blocking solution for 1 h in a 37 °C humidified chamber. The blocking solution was discarded, and the slides were incubated with target antibodies (ALK5 and MARCH2) diluted in Duolink® antibody diluent at 37 °C for 2 h in a humidified chamber. The slides were washed twice with Duolink® wash buffer A for 5 min each at room temperature. PLA probes, diluted 1:5 in Duolink® antibody diluent, were added to the slides and incubated in a 37 °C humidified chamber for 1 h. The slides were washed again twice with Duolink® wash buffer A for 5 minutes each at room temperature. Next, the slides were incubated with ligase, diluted 1:40 in Duolink® dilution buffer, at 37 °C for 30 min in a humidified chamber. The slides were washed twice with Duolink® wash buffer A for 5 min each at room temperature. Then, the slides were incubated with polymerase, diluted 1:80 in Duolink® amplification buffer, at 37 °C for 100 min in a humidified chamber. The slides were washed twice with Duolink® wash buffer B for 5 min each at room temperature and mounted with a minimal volume of Duolink® PLA mounting solution containing DAPI. After incubation at room temperature for 15 min, fluorescence analysis was performed using a confocal microscope. Fluorescence microscopy was carried out using a Zeiss LSM 700 confocal microscope and ZEN software.

### Isolation of plasma membrane proteins

Cells were cultured in 60 mm plates and rinsed three times with ice-cold PBS. The cells were then incubated with PBS containing 1 mg/mL of sulfo-NHS-biotin (APExBIO, Houston, TX, USA) for 20 min. The reaction was quenched with PBS containing 150 mM glycine, followed by an additional three washes. Cells were then harvested and lysed in lysis buffer (1% NP-40, 20 mM Tris–HCl, pH 7.6, 150 mM NaCl, 2 mM EDTA, 10 mM NaF, 1 mM Na_3_VO_4_, and a protease inhibitor cocktail). After lysis, the lysates were centrifuged at 13,000 rpm for 15 min. The supernatant was transferred to a new tube and incubated with streptavidin magnetic beads (Thermo Scientific, Waltham, MA, USA, #88816) for 3 h. The immunoprecipitates were washed five times with lysis buffer and subsequently eluted using 2.5× Laemmli sample buffer.

### Endosome isolation

The LysoIP method was modified to isolate endosomes [57]. A549 cells stably expressing FLAG-Rab5 and HA-MARCH2 were rinsed with PBS and then scraped into 1 ml of PBS containing a protease inhibitor cocktail. The cells were mechanically disrupted by ten passages through a 26 G needle. The lysate was then centrifuged at 1000 x g for 2 min at 4 °C, and the supernatant, containing cellular organelles, was incubated with anti-FLAG magnetic agarose on a rotator for 15 min. The immunoprecipitates were washed three times with PBS, and the beads containing endosomes were incubated with 1% Triton X-100 lysis buffer for 10 min. The beads were removed by magnetic separation, and the eluted endosome fraction was used for further analysis.

### Immunoprecipitation (IP) kinase assay

Cell lysates were prepared in lysis buffer (1% NP-40, 20 mM Tris–HCl, pH 7.6, 150 mM NaCl, 2 mM EDTA, 10 mM NaF, 1 mM Na_3_VO_4_, and a protease inhibitor cocktail) and incubated with anti-Flag magnetic agarose (A36797) at 4 °C for 2 h. The bead-protein complex was washed five times with lysis buffer. The bead-protein complex was resuspended in 50 µl of kinase buffer (25 mM Tris, pH 7.5, 5 mM β-Glycerophosphate, 2 mM DTT, 0.1 mM Na_3_VO_4_, and 10 mM MgCl_2_). GST-SMAD2 was added and incubated for 30 min at 30 °C. The reaction was terminated by adding 5x sample buffer and analyzed by immunoblotting.

### Wound healing assay

Cells were cultured in a 6-well plate and transfected with the indicated siRNA. After 24 h, a scratch wound was generated using the SPLScar^TM^ Scratcher (SPL, Cat. 201906). The wounded cell layers were washed once with culture medium and subsequently incubated in culture medium with or without TGF-β. The wound area was captured at each designated time point after scratching and quantified using ImageJ software. Each experiment was repeated three times.

### Transwell invasion assay

Transwell plates (6.5 mm Transwell inserts with an 8.0 µm pore polycarbonate membrane; Corning Costar, Kennebunk, ME, USA) were used for the Transwell invasion assay. Cells (0.5 × 10^5^) in 200 µL of 0.1% FBS culture medium were seeded in the upper chamber, and 600 µL of 10% FBS culture medium with or without TGF-β was placed in the lower chamber. Following incubation for 16 h, cells were fixed with 3% paraformaldehyde for 10 min and permeabilized with 0.1% Triton X-100 for 5 min. Cells were stained with hematoxylin, and non-migrated cells adhering to the upper chamber were removed using a cotton swab. Stained cells were counted under a light microscope. Each experiment was repeated three times.

### Mass spectrometry analysis

Excised gel bands were chopped into smaller pieces and subsequently destained with 100 mM ammonium bicarbonate/acetonitrile (1:1, v/v). Following destaining, in-gel digestion with trypsin was performed [58]. After digestion, the extracted peptides from the gel pieces were analyzed using a Q-Exactive mass spectrometer coupled with an UltiMate 3000 HPLC system (Thermo Fisher Scientific). In this LC-MS system, peptides were separated on an EASY-Spray Column (15 cm × 75 µm I.D., C18, particle size of 2 µm for mapping ubiquitination sites at ALK5, or 50 cm × 75 µm I.D., C18, particle size of 2 µm for identifying the interactome with HA-tagged MARCH2). Mobile phases A and B consisted of 0.1% (v/v) formic acid and 0.1% (v/v) formic acid in 80% (v/v) acetonitrile, respectively. The mass spectrometer parameters were set as previously described [59]. Mass spectra were analyzed using the SEQUEST HT module of Proteome Discoverer 2.4 (Thermo Fisher Scientific). The human proteome database was downloaded from Uniprot (20,515 protein entries, Release 2023_03), and the MS data were searched against the human proteome database, including a list of commonly encountered protein contaminants from the Common Repository of Adventitious Proteins (cRAP, downloaded from http://www.thegpm.org/crap). The mass tolerance values were set to 10 ppm for precursor ions and 0.02 Da for fragment ions. The search parameters included full tryptic specificity with up to two missed cleavages and carbamidomethylation ( + 57.021 Da) at cysteine as a static modification. Variable modifications included methionine oxidation ( + 15.995 Da) and acetylation at the protein N-terminus ( + 42.011 Da). For identifying ubiquitination sites at ALK5, diGly modification at lysine ( + 114.043 Da) was additionally included as a dynamic modification. The false discovery rate (FDR) was set to 0.01 at both the peptide and the peptide spectrum match (PSM) levels using the Percolator module.

### Tumor metastasis mouse model

Six-week-old male C57BL/6 N mice were purchased from DBL (Chungcheongbuk-do, Eumseong, Republic of Korea). All animal experimental procedures were approved by the Institutional Animal Care and Use Committee (IACUC) of the Sungkyunkwan University. Mice were randomly assigned to the following groups: 1) Normal, 2) Wild-type (WT) TC-1, 3) MARCH2 knockout (KO) TC-1 4) ALK5 KO TC-1, 5) ALK5 KO + ALK5 WT TC-1, and 6) ALK5 KO + ALK5 K342/343 R TC-1. TC-1 and other modified TC-1 cell lines were cultured and injected intravenously into C57BL/6 N mice at a concentration of 3 × 10^5^ cells, diluted in 200 μL of PBS. Mouse body weight was monitored every two days, starting from the day of TC-1 injection. The mice were sacrificed 18 days post-injection, and lung tissues were isolated and fixed in 4% paraformaldehyde. The lung tissue sections were stained with hematoxylin and eosin. Stained slides were examined under a microscope at 40x and 100x magnifications. No blinding was performed during the experiment or outcome assessment. However, objective measures were used to reduce potential bias.

### Subcutaneous tumor model

Mice were randomly assigned, and TC-1 or TC-1 MARCH KO cells (1 × 10⁶) were subcutaneously injected into C57BL/6 N mice together with Matrigel. Tumor volume was calculated using the following formula: (length × width²) × 0.5. No blinding was performed during the experiment or outcome assessment. However, objective measures were used to reduce potential bias.

### Statistical analysis

Appropriate statistical tests were selected based on the experimental design. Statistical significance was determined using a two-tailed unpaired *t*-test to compare differences between two groups. These analyses were performed using GraphPad Prism 5 and SPSS version 18. *P*-values < 0.05 were considered statistically significant. Survival analysis was conducted using the Kaplan–Meier (KM) Plotter Tool, and statistical significance was assessed using the log-rank test. All experiments were repeated at least three times. The *n* number for in vivo mouse experiments is indicated in the figure legends. The sample size was chosen based on the literature in the field, aiming to balance ethical considerations with statistical requirements. No statistical method was used to predetermine the sample size.

## Supplementary information

Supplemental Information

Mass spectrometry of MARCH2-interacting proteins

Uncropped WB image

## Data Availability

The mass spectrometry proteomics data have been deposited in the Korea BioData Station (K-BDS) with the K-BDS BioProject Number KAP240689.
