# Supplementary material for: MARCH2-mediated Lys63-linked polyubiquitination promotes metastasis by modulating the catalytic activity of TGF-β type I receptor
Source: Cell Death Dis. 2025 Nov 10;16(1):814. doi: 10.1038/s41419-025-08145-3 (PMC12603192; doi:10.1038/s41419-025-08145-3)
Supplement: Supplementary file 1 — Supplemental Information [file 41419_2025_8145_MOESM1_ESM.docx]

**Supplemental information**

MARCH2-mediated Lys63-linked polyubiquitination promotes metastasis by modulating the catalytic activity of TGF-β type I receptor

Kun Tae, Sang Woo Cho, Seonjeong Lee, Dahyoon Heo, Hyo Sun Cha, Da Yeon Lee, Eunjeong Oh, Minhyeong Choi, Donghyuk Shin, Siyoung Yang, Cheolju Lee, and Cheol Yong Choi


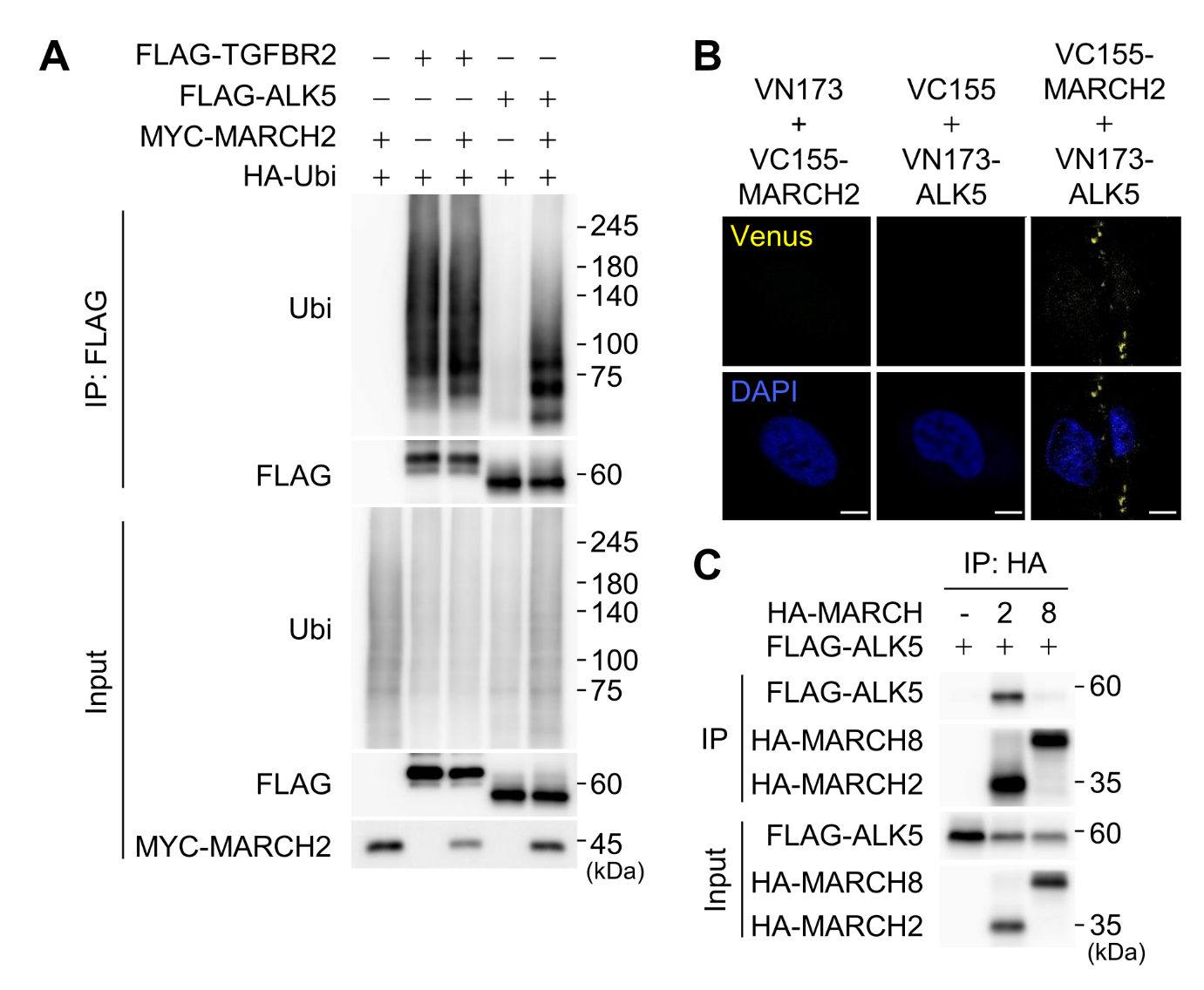


**Figure S1. MARCH2 does not ubiquitinate TGFBR2 and ALK5 does not interact with MARCH8. A** A ubiquitination assay was performed to compare the ubiquitination of ALK5 and TGFBR2. HEK293T cells were co-transfected with FLAG-ALK5, FLAG-TGFBR2, HA-ubiquitin, and MYC-MARCH2. Cell lysates containing ubiquitin-conjugated FLAG-ALK5 and FLAG-TGFBR2, from cells co-transfected with or without MYC-MARCH2, were analyzed by immunoblotting with the indicated antibodies. **B** A bimolecular fluorescence complementation (BiFC) assay was performed to assess the interaction between MARCH2 and ALK5 in live cells. HeLa cells were transfected with either VC155-MARCH2 or VN173-ALK5 alone, or with both VC155-MARCH2 and VN173-ALK5. Yellow fluorescence was detected in cells co-expressing VC155-MARCH2 and VN173-ALK5. Scale bar: 10 μm. **C** A co-immunoprecipitation assay was performed to examine the interaction of FLAG-ALK5 with either HA-MARCH2 or HA-MARCH8. HEK293T cells were co-transfected with FLAG-ALK5 and either HA-MARCH2 or HA-MARCH8. Proteins immunoprecipitated with HA-MARCH2 or HA-MARCH8 were analyzed by immunoblotting with anti-FLAG and anti-HA antibodies.


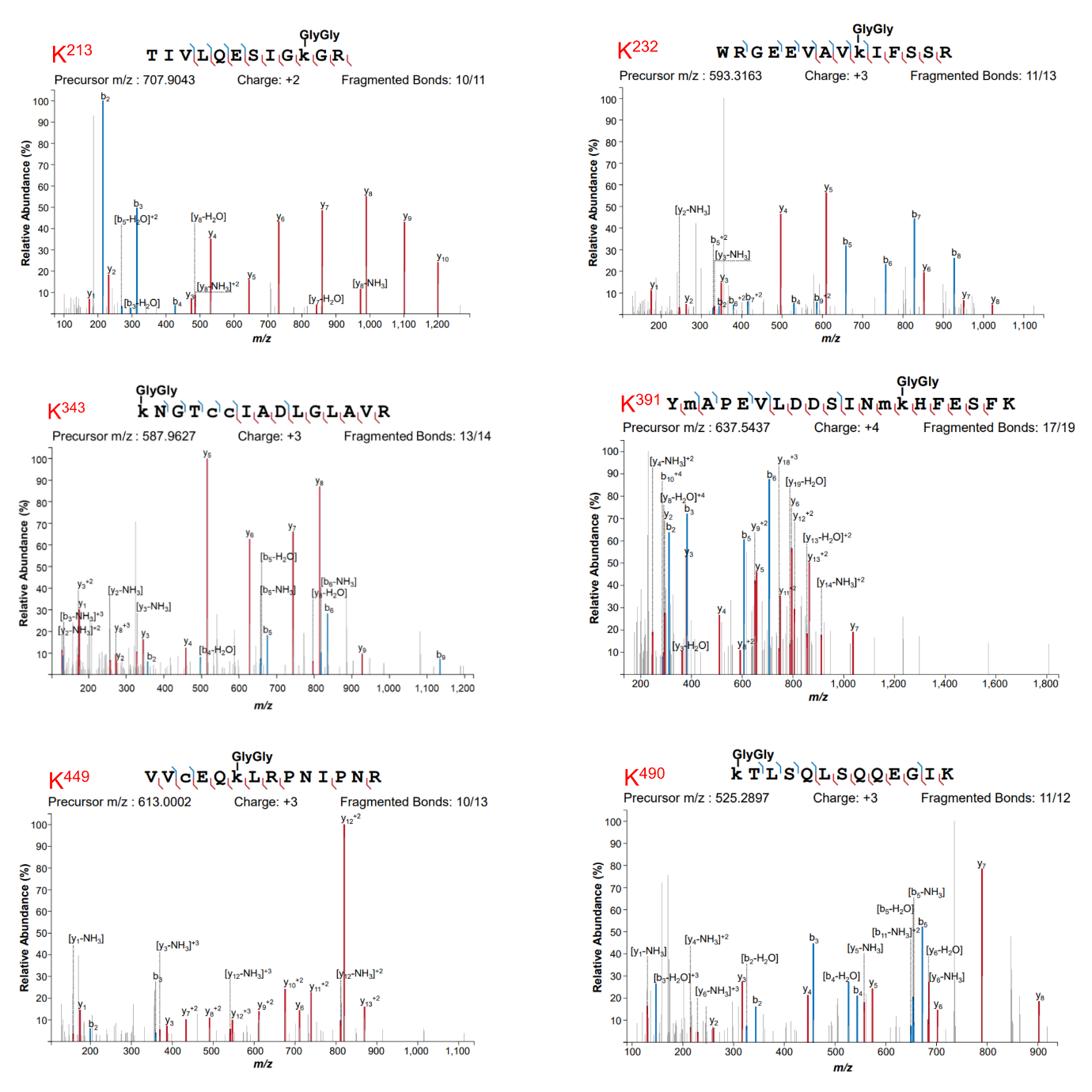


**Figure S2. Mass spectrometry analysis of MARCH2-mediated ALK5 ubiquitination.**

The mass spectra of ubiquitinated ALK5 peptides generated by MARCH2 are presented. Peaks corresponding to the expected singly- and doubly-charged b- and y-ions are labeled. Ubiquitinated lysine residues are indicated with their positions and denoted as K-GlyGly.


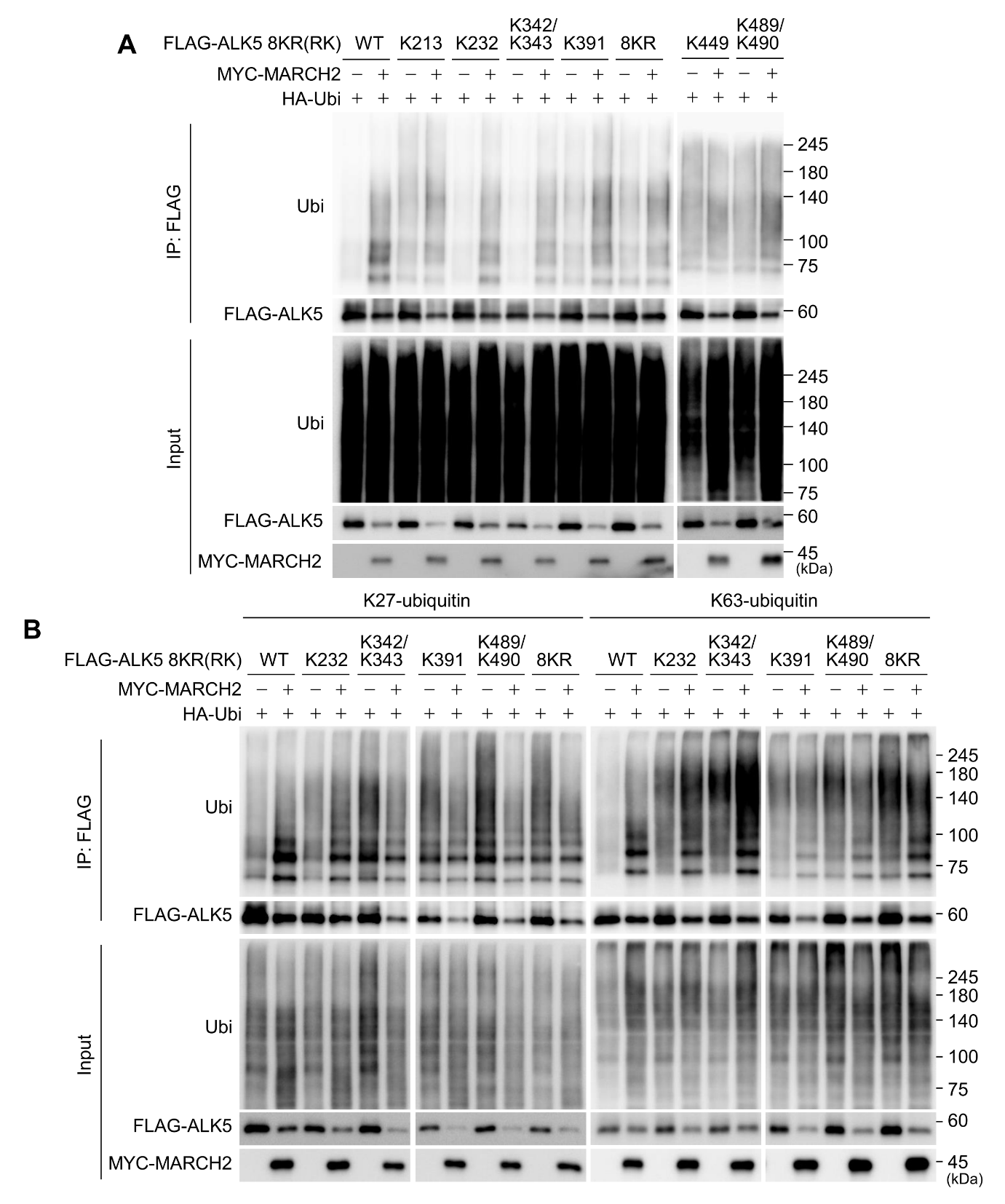


**Figure S3. ALK5 ubiquitination via K27- and K63-linked chains at multiple lysine residues. A** A ubiquitination assay of ALK5 was performed using an ALK5 mutant in which one or two individual lysine residues were restored in the 8KR mutant (K213R, K232R, K342/343R, K391R, K449R, and K489/490R). HEK293T cells were co-transfected with HA-Ubiquitin, MYC-MARCH2, and individual FLAG-ALK5 RK mutants. Ubiquitin-conjugated FLAG-ALK5 was analyzed by immunoblotting with the indicated antibodies. **B** A ubiquitination assay of ALK5 was performed using an ALK5 mutant in which one or two individual lysine residues were restored in the 8KR mutant. HEK293T cells were co-transfected with HA-Ubiquitin (K27 or K63), MYC-MARCH2, and individual FLAG-ALK5 RK mutants. Ubiquitin-conjugated FLAG-ALK5 was analyzed by immunoblotting with the indicated antibodies.


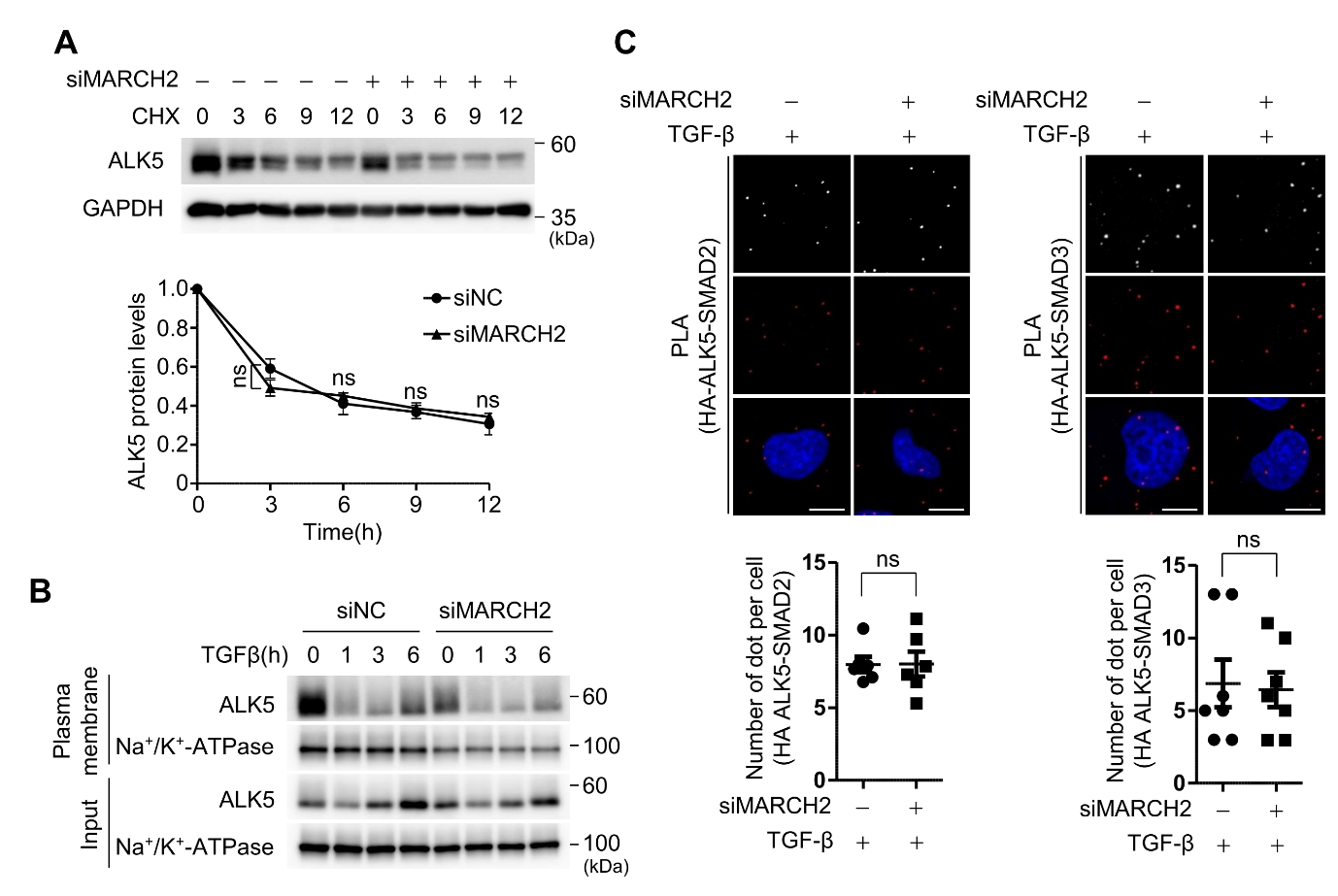


**Figure S4. MARCH2 does not affect ALK5 abundance, internalization, or interaction with SMAD2. A** A cycloheximide chase assay was performed to assess ALK5 stability in MARCH2-depleted cells. A549 cells were transfected with either mock or MARCH2-targeting siRNA and treated with cycloheximide (10 mg/ml) for the indicated time points. ALK5 levels were quantified and are shown in the graph. **B** The levels of ALK5 in the plasma membrane fraction were determined. A549 cells transfected with either mock or MARCH2-targeting siRNA were treated with TGF-β for the indicated durations. Biotinylated plasma membrane fractions were isolated using streptavidin-agarose beads. ALK5 levels in total lysates and plasma membrane fractions were analyzed by immunoblotting with the indicated antibodies. Na^+^/K^+^-ATPase was used as a positive control for the plasma membrane fraction. **C** A proximity ligation assay (PLA) was performed to visualize the interaction between HA-ALK5 and SMAD2 (left panel) or SMAD3 (right panel) following TGF-β treatment. A549 cells stably expressing HA-ALK5 were transfected with either mock or MARCH2-targeting siRNA and treated with TGF-β for 1 hour. Interactions were visualized using Texas Red fluorescence and analyzed by confocal microscopy. Fluorescent puncta were quantified using ImageJ software, and red fluorescence was converted to white for clarity. The number of puncta is presented in the graph below. Scale bar: 10 μm. Statistical analysis for (**C**) was performed using a two-tailed unpaired *t*-test, and data are presented as the mean ± SD, ns (not significant).


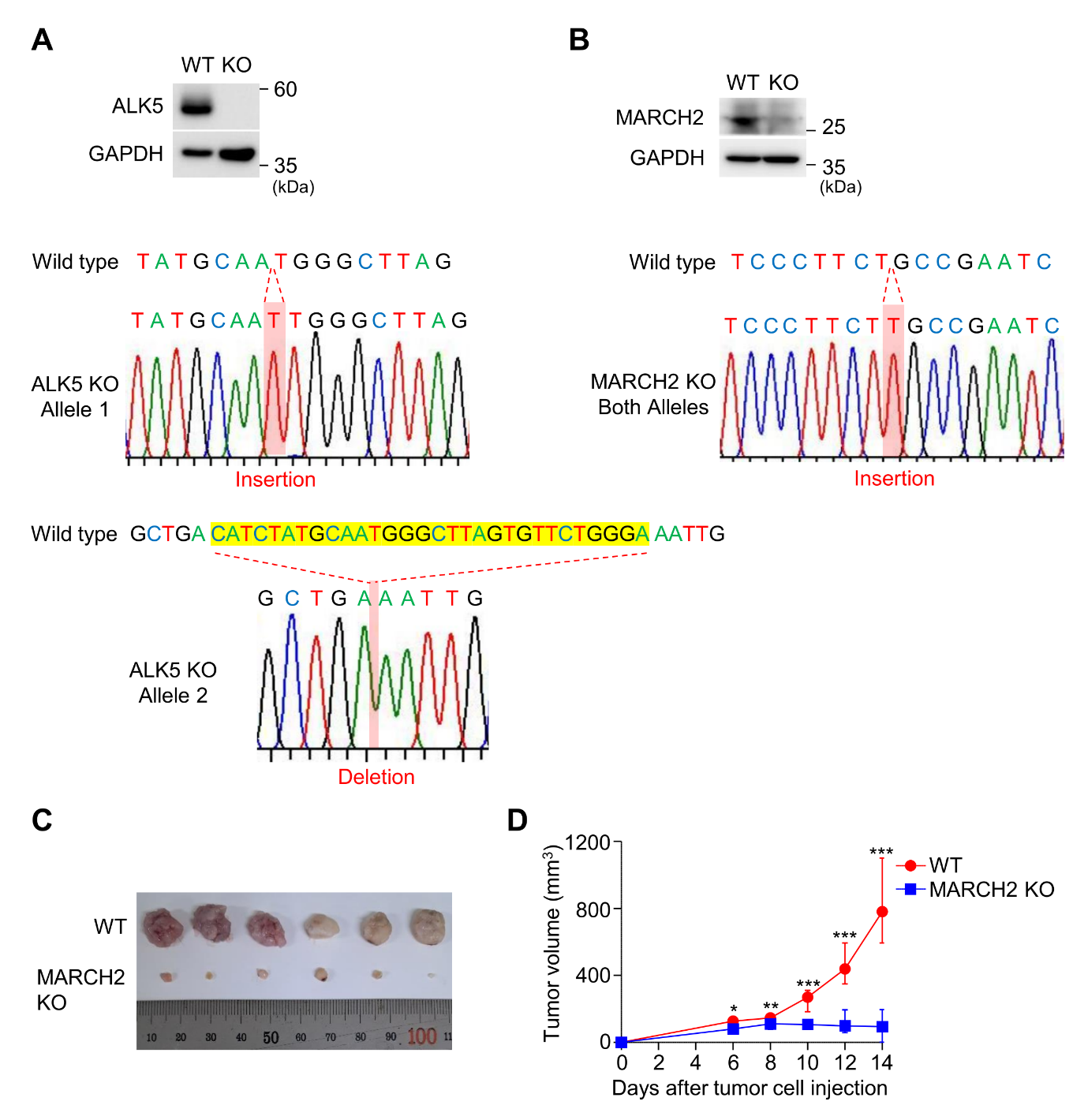


**Figure S5. Generation of ALK5 and MARCH2 knockout cells. A** ALK5 knockout (KO) TC-1 cells were generated using the CRISPR-Cas9 system. Immunoblotting with an anti-ALK5 antibody confirmed the absence of ALK5 expression in the KO cells. Sequencing of the ALK5 alleles revealed that the open reading frame was disrupted by a single T insertion in one allele and a 30-nucleotide deletion in the other. **B** MARCH2 KO TC-1 cells were generated using the CRISPR-Cas9 system. Immunoblotting with an anti-MARCH2 antibody confirmed the absence of MARCH2 expression in MARCH2 KO cells. Sequencing of the MARCH2 alleles from KO cells revealed that the open reading frame was disrupted by a single T insertion in both alleles. **C** Representative tumor samples from WT and MARCH2 KO groups are shown. **D** Growth curves of subcutaneous TC-1 tumors in mice injected with WT or MARCH2 KO TC-1 cells are presented. Tumor volumes (mm^3^) were measured every one or two days (n=6). Statistical analysis for (**D**) was performed using a two-tailed unpaired *t*-test, and data are presented as the mean ± SD, where **p* < 0.05, ***p* < 0.01, ****p* < 0.001.


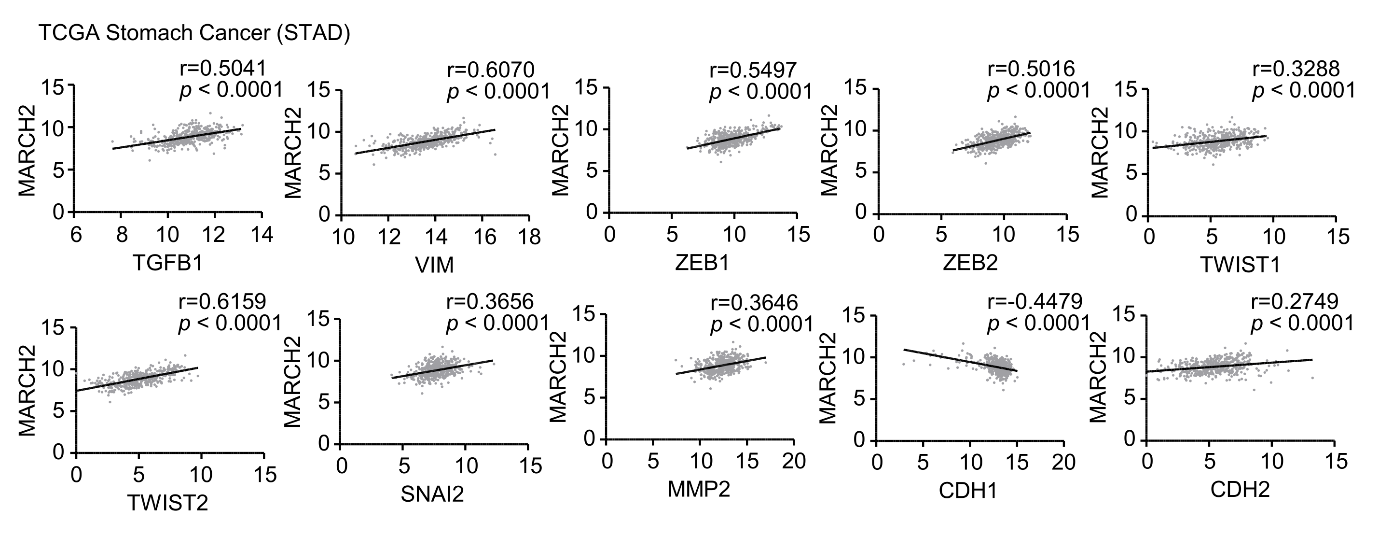


**Figure S6. Gene expression correlation analysis of MARCH2 and TGF-β target genes.**

The correlation between MARCH2 mRNA levels and TGF-β target genes in human stomach cancer patients was analyzed using TCGA datasets. MARCH2 gene expression levels were compared with those of TGF-β target genes, including TGFB1, VIM, ZEB1, ZEB2, TWIST1, TWIST2, SNAI2, MMP2, CDH1, and CDH2. The Pearson correlation coefficient (r) and corresponding *p* values are shown for each analysis.


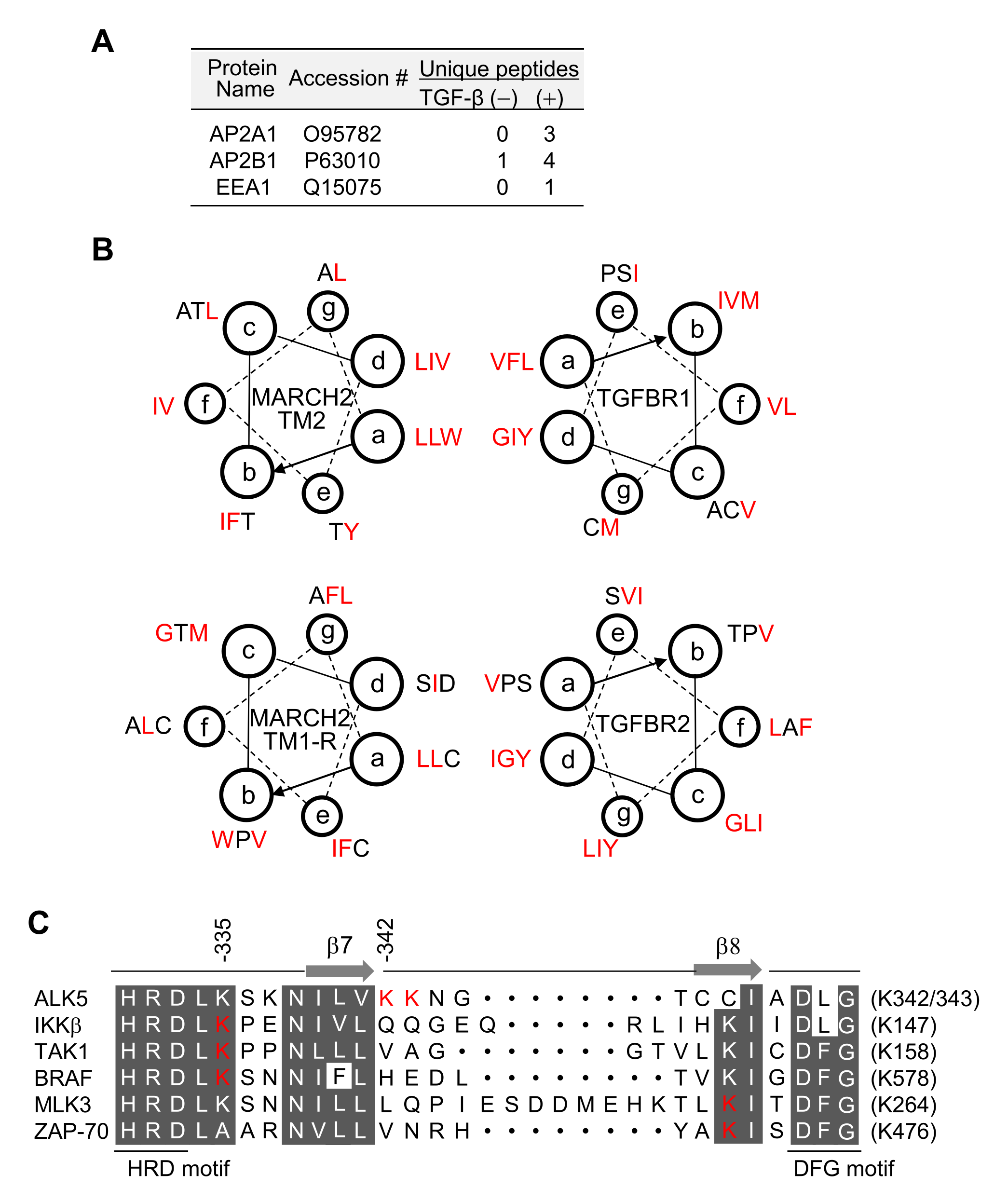


**Figure S7. Helical wheel diagrams of transmembrane domains and amino acid alignment of catalytic loops in protein kinases. A** MARCH2-interacting proteins were identified via mass spectrometry. Proteins involved in clathrin-mediated endocytosis are shown. AP2A1 and EEA1 were detected exclusively under TGF-β treatment, while AP2B1 interaction was enhanced in the presence of TGF-β. **B** Helical wheel diagrams of the transmembrane domains of MARCH2 TM1, MARCH2 TM2, TGFBR2, and TGFBR1 (ALK5) are shown. The heptad repeat is displayed as positions a to g. Amino acids associated with TM-TM interactions, including hydrophobic residues (L, I, and V), aromatic residues (W, F, and Y), and glycine (G), are highlighted in red. **C** Alignment of the amino acid sequences of the catalytic loops of ALK5 and other protein kinases, including IKKβ, TAK1, BRAF, MLK3, and ZAP-70. The alignment highlights sequence identity and similarity (indicated in black) across all sequences. The secondary structural elements of ALK5 and amino acid positions are indicated above the alignment. The lysine residue conjugated to K63-linked ubiquitin chains, which influences catalytic activity, is highlighted in red. The alignment was generated using CLUSTALW.
