# Supplementary material for: MARCH2-mediated Lys63-linked polyubiquitination promotes metastasis by modulating the catalytic activity of TGF-β type I receptor
Source: Cell Death Dis. 2025 Nov 10;16(1):814. doi: 10.1038/s41419-025-08145-3 (PMC12603192; doi:10.1038/s41419-025-08145-3)

Fig. 1A

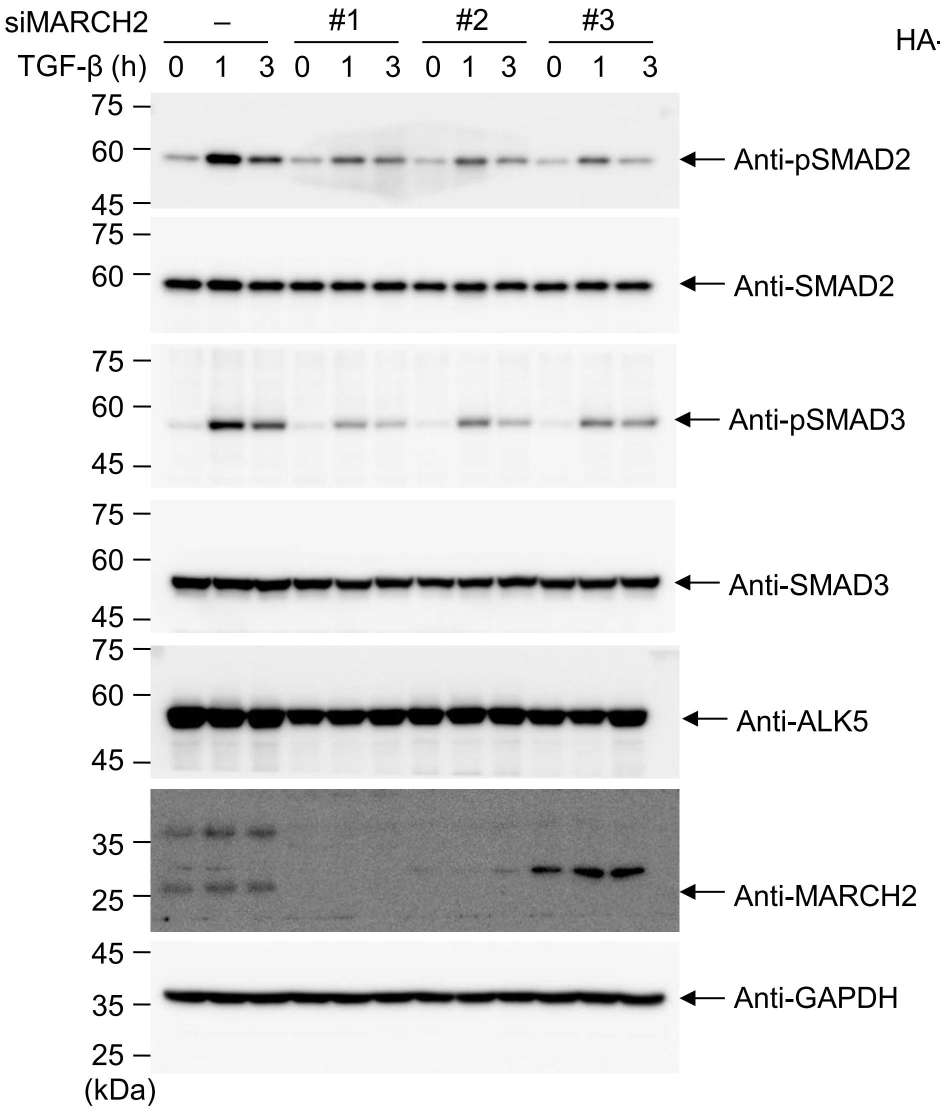

Fig. 1C

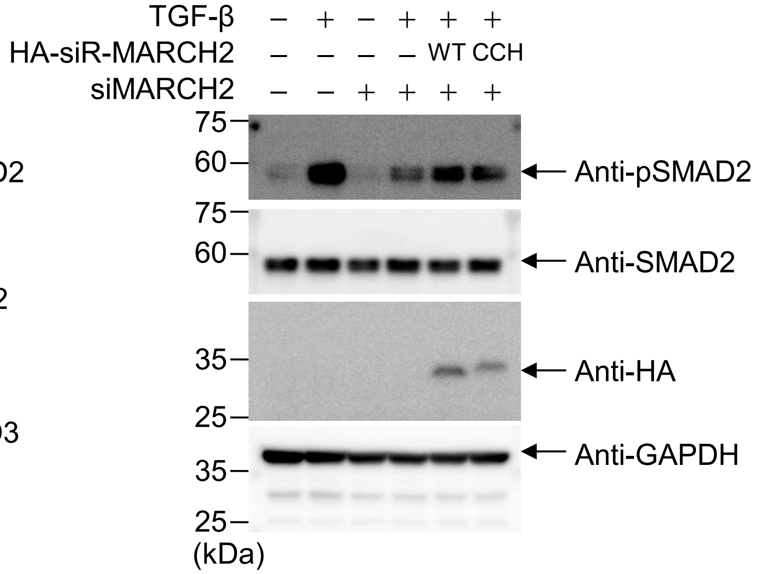

Fig. 1D

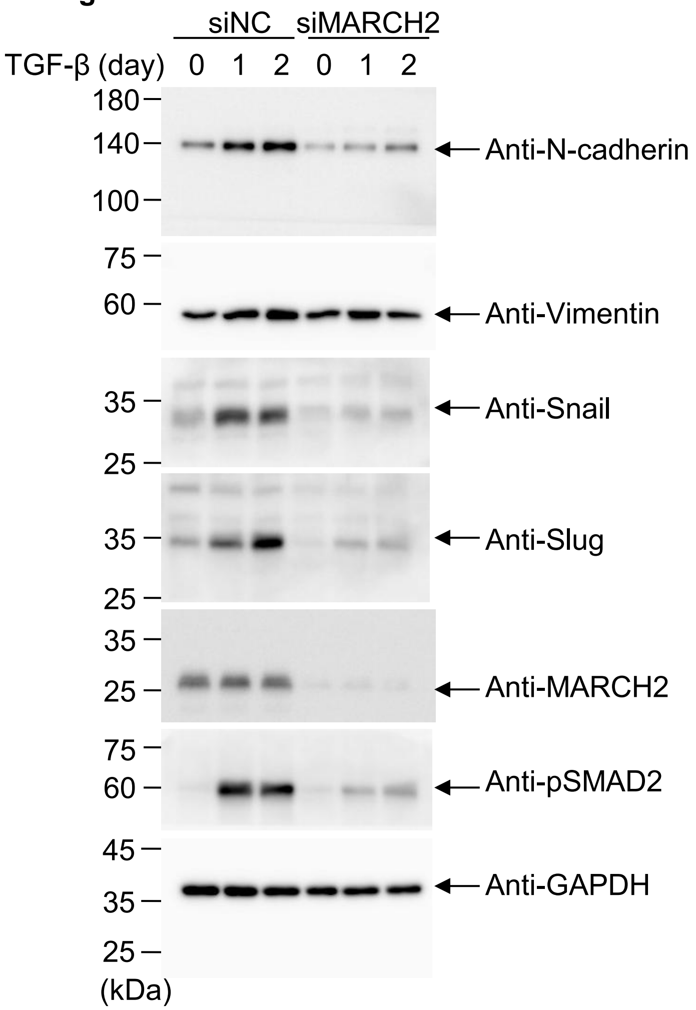

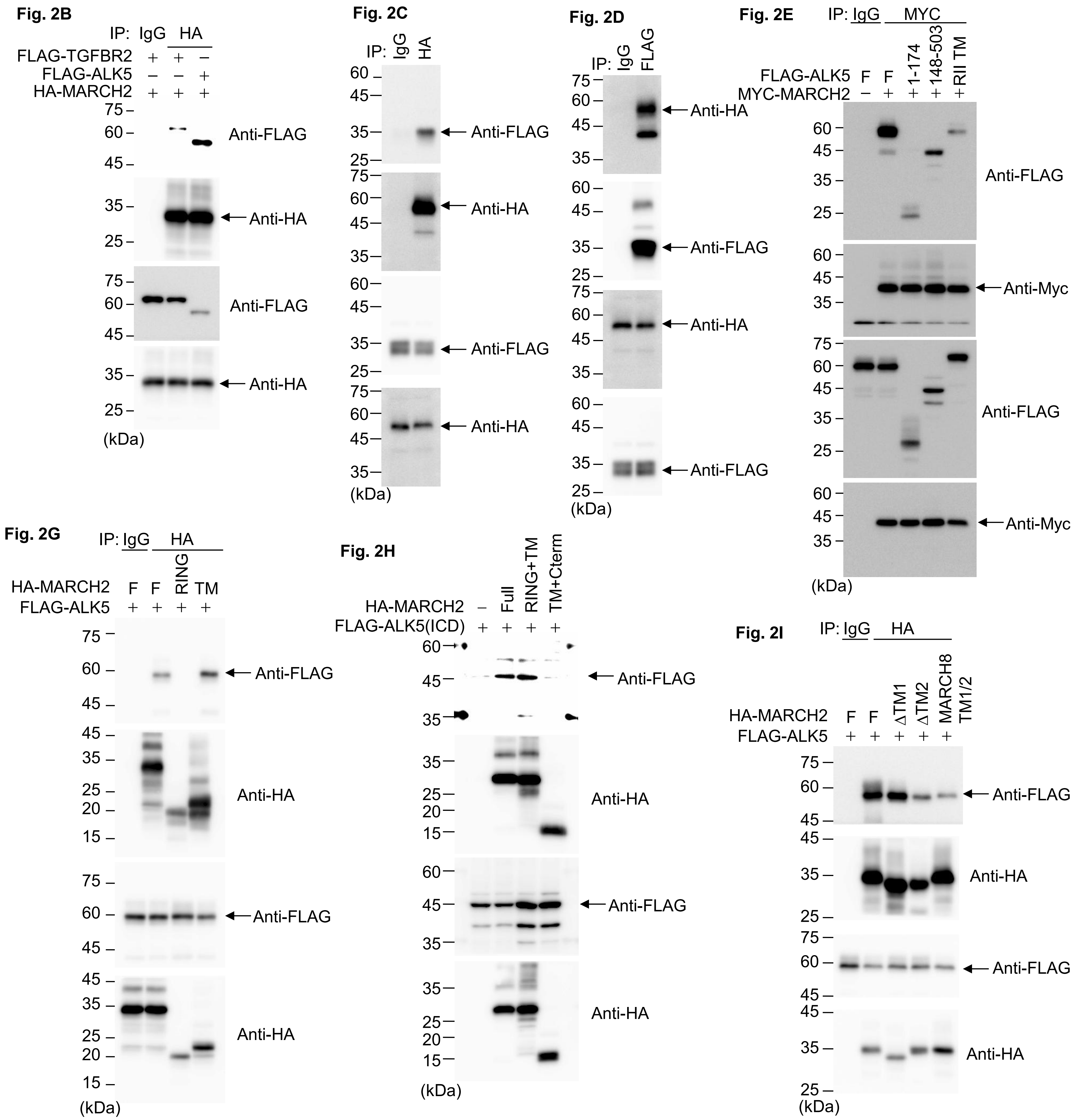
Fig. 2B
IP: IgG HA
FLAG-TGFBR2 + + −
FLAG-ALK5 − − +
HA-MARCH2 + + +
Anti-FLAG
Anti-HA
Anti-FLAG
Anti-HA
(kDa)
Fig. 2C
IP: IgG HA
Anti-FLAG
Anti-HA
Anti-FLAG
Anti-HA
(kDa)
Fig. 2D
IP: IgG FLAG
Anti-HA
Anti-FLAG
Anti-HA
Anti-FLAG
(kDa)
Fig. 2E
IP: IgG MYC
FLAG-ALK5 F F 1-174 148-503 RII TM
MYC-MARCH2 − + + + +
Anti-FLAG
Anti-Myc
Anti-FLAG
Anti-Myc
(kDa)
Fig. 2G
IP: IgG HA
HA-MARCH2 F F RING TM
FLAG-ALK5 + + + +
Anti-FLAG
Anti-HA
Anti-FLAG
Anti-HA
(kDa)
Fig. 2H
HA-MARCH2 − Full RING+TM TM+Cterm
FLAG-ALK5(ICD) + + + +
Anti-FLAG
Anti-HA
Anti-FLAG
Anti-HA
(kDa)
Fig. 2I
IP: IgG HA
HA-MARCH2 F F ΔTM1 ΔTM2 MARCH8 TM1/2
FLAG-ALK5 + + + + +
Anti-FLAG
Anti-HA
Anti-FLAG
Anti-HA
(kDa)

Fig. 3A

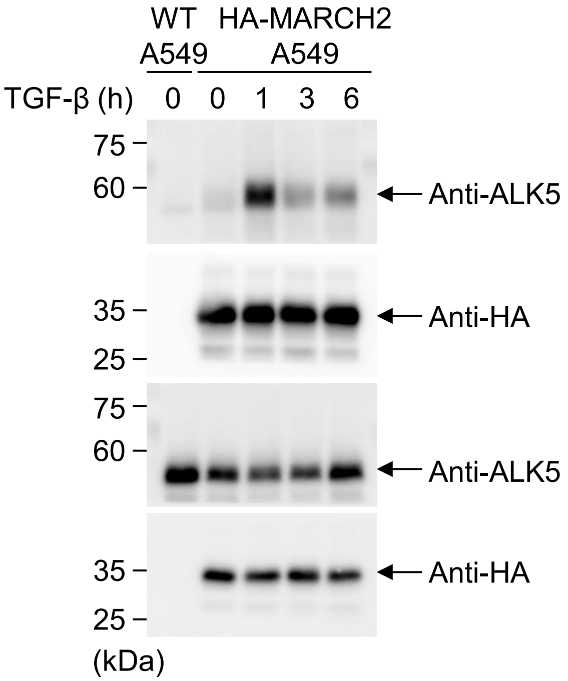

Fig. 3B

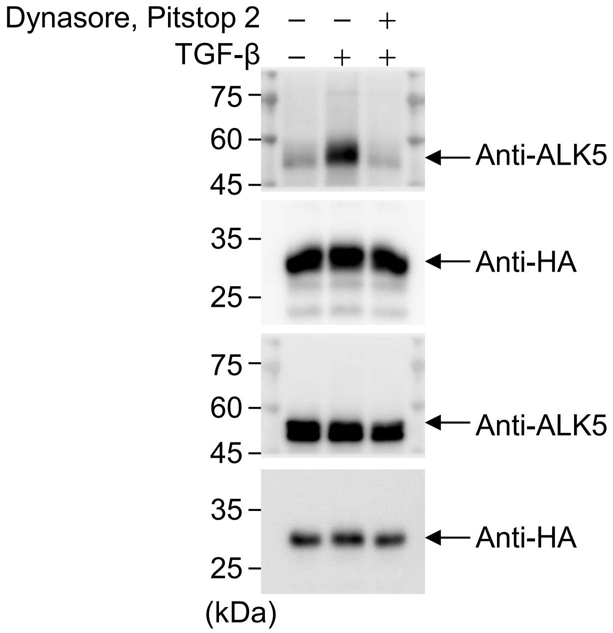

Fig. 3D

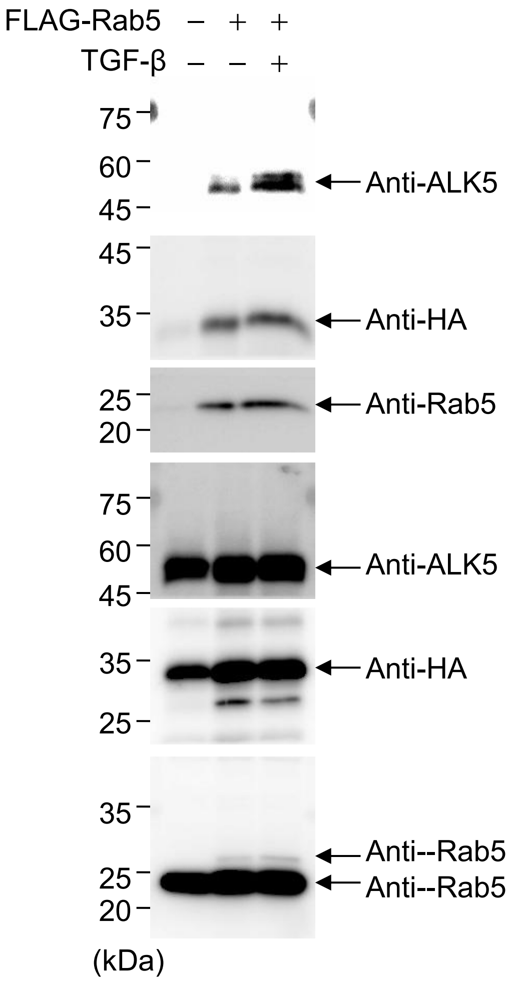

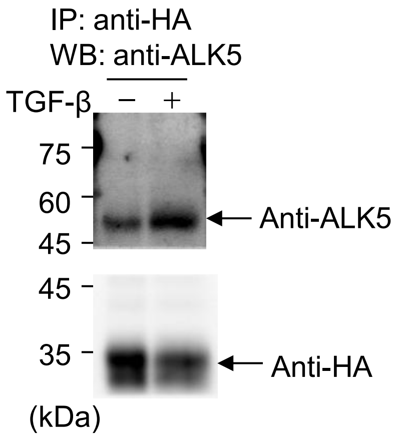

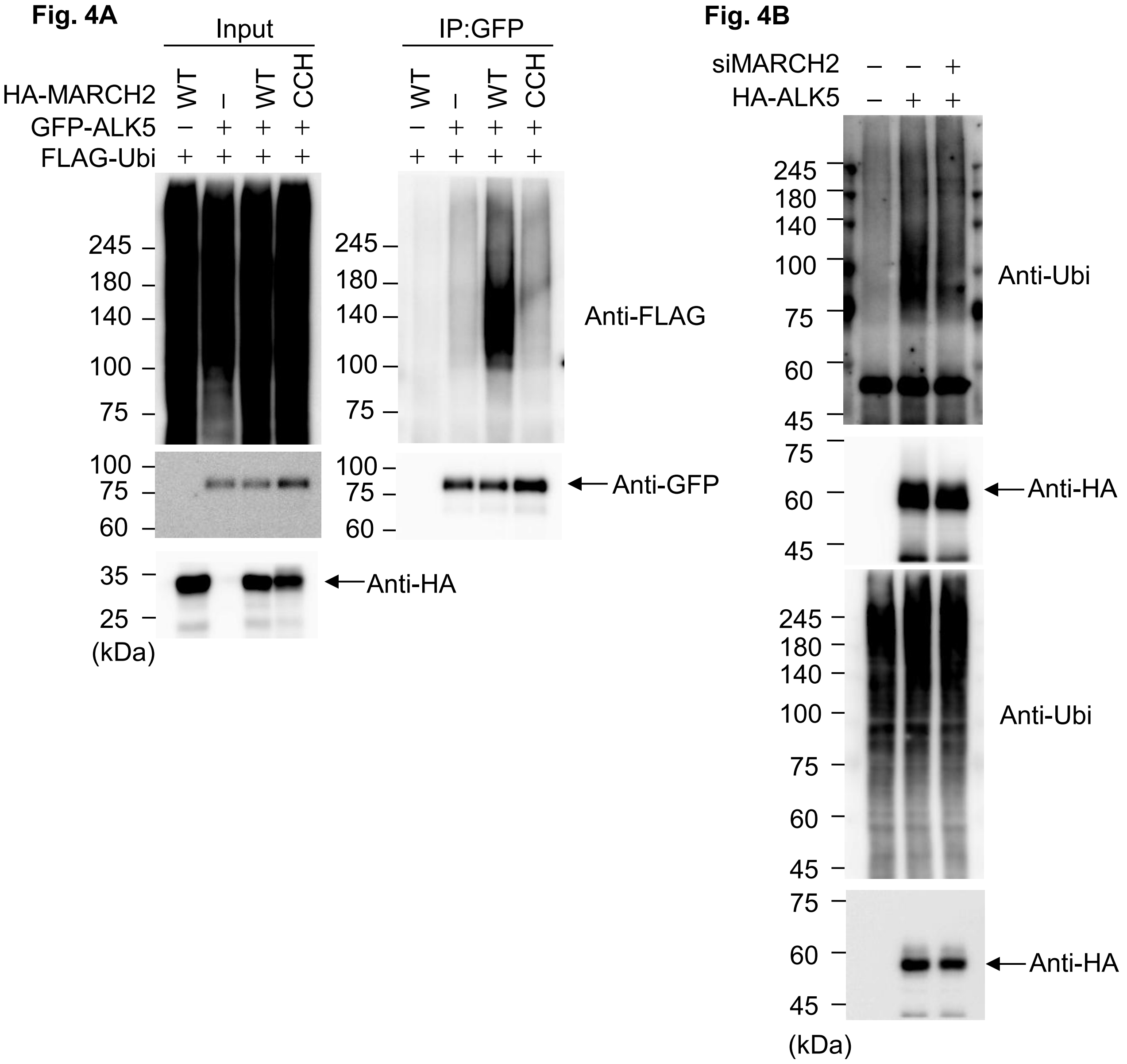

Fig. 4A
Input
IP:GFP
HA-MARCH2
WT
–
WT
CCH
GFP-ALK5
FLAG-Ubi
245
180
140
100
75
60
35
25
(kDa)
Anti-FLAG
Anti-GFP
Anti-HA
Fig. 4B
siMARCH2
HA-ALK5
45
Anti-Ubi
Anti-HA

Fig. 4D

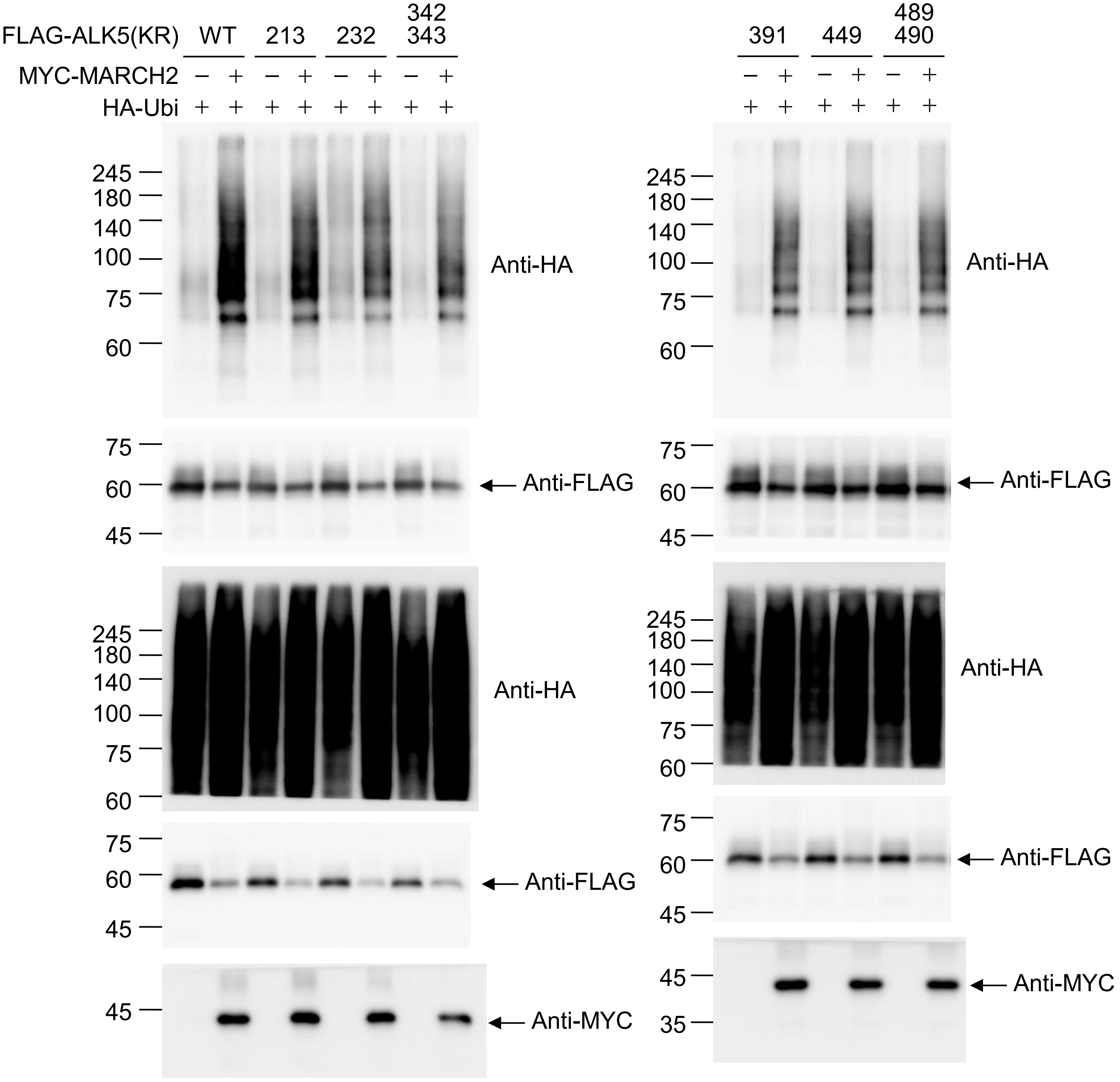
FLAG-ALK5(KR)
WT
213
232
342 343
391
449
489 490
MYC-MARCH2
HA-Ubi
245
180
140
100
75
60
45
35
Anti-HA
Anti-FLAG
Anti-MYC

Fig. 4E

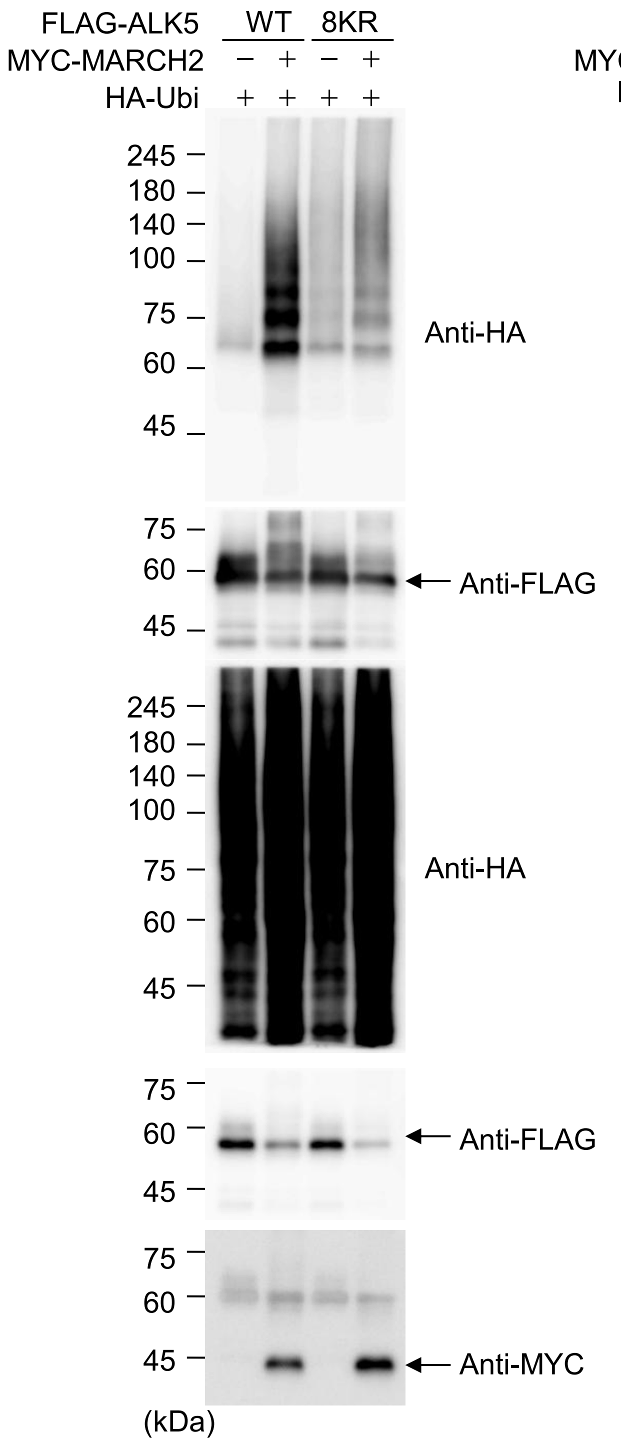

FLAG-ALK5
WT
8KR
MYC-MARCH2
HA-Ubi
245
180
140
100
75
60
45
Anti-HA
Anti-FLAG
Anti-MYC
(kDa)

Fig. 4F

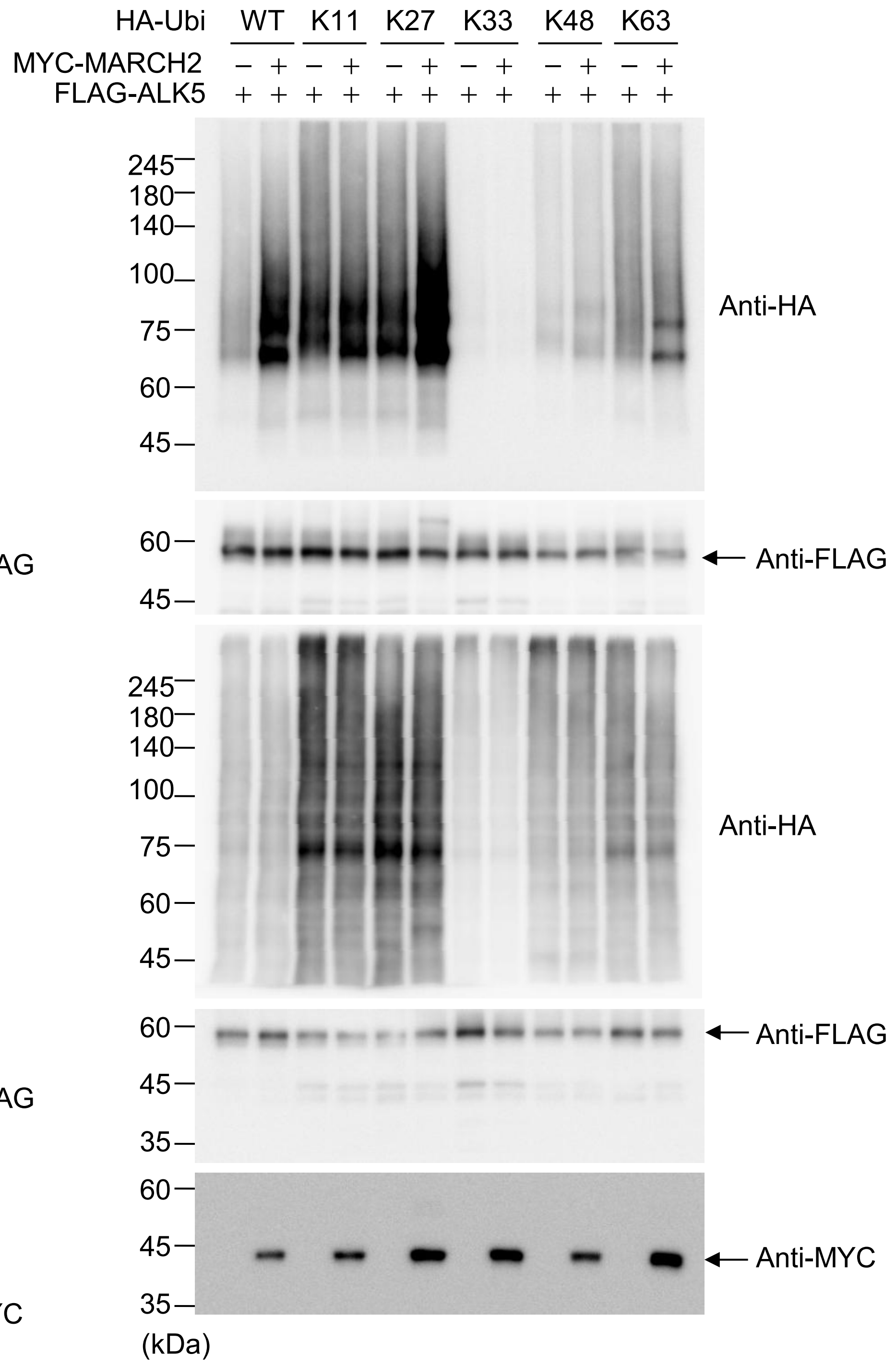

HA-Ubi
WT
K11
K27
K33
K48
K63
MYC-MARCH2
FLAG-ALK5
245
180
140
100
75
60
45
35
Anti-HA
Anti-FLAG
Anti-MYC
(kDa)

Fig. 4G

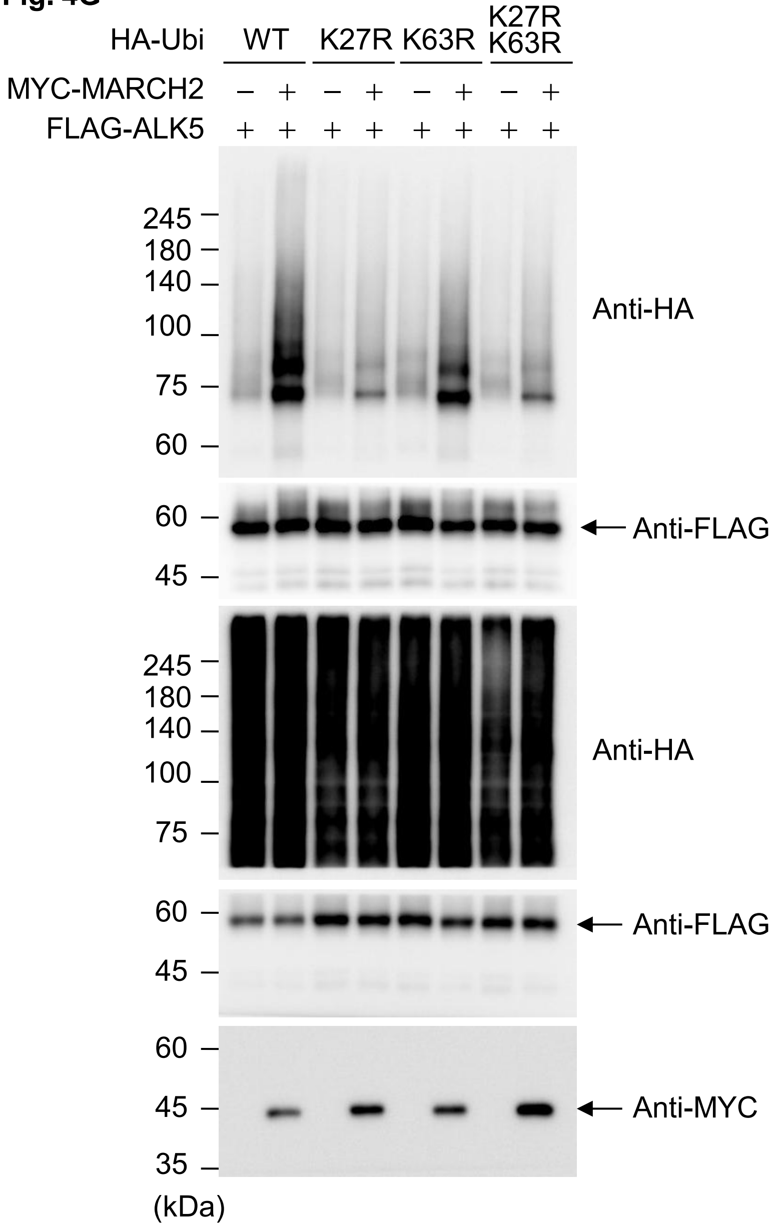

HA-Ubi
WT
K27R
K63R
K27R K63R
MYC-MARCH2
– + – + – + – +
FLAG-ALK5
+ + + + + + + +
245
180
140
100
75
60
Anti-HA
60
45
Anti-FLAG
245
180
140
100
75
Anti-HA
60
45
Anti-FLAG
60
45
35
Anti-MYC
(kDa)

Fig. 4H

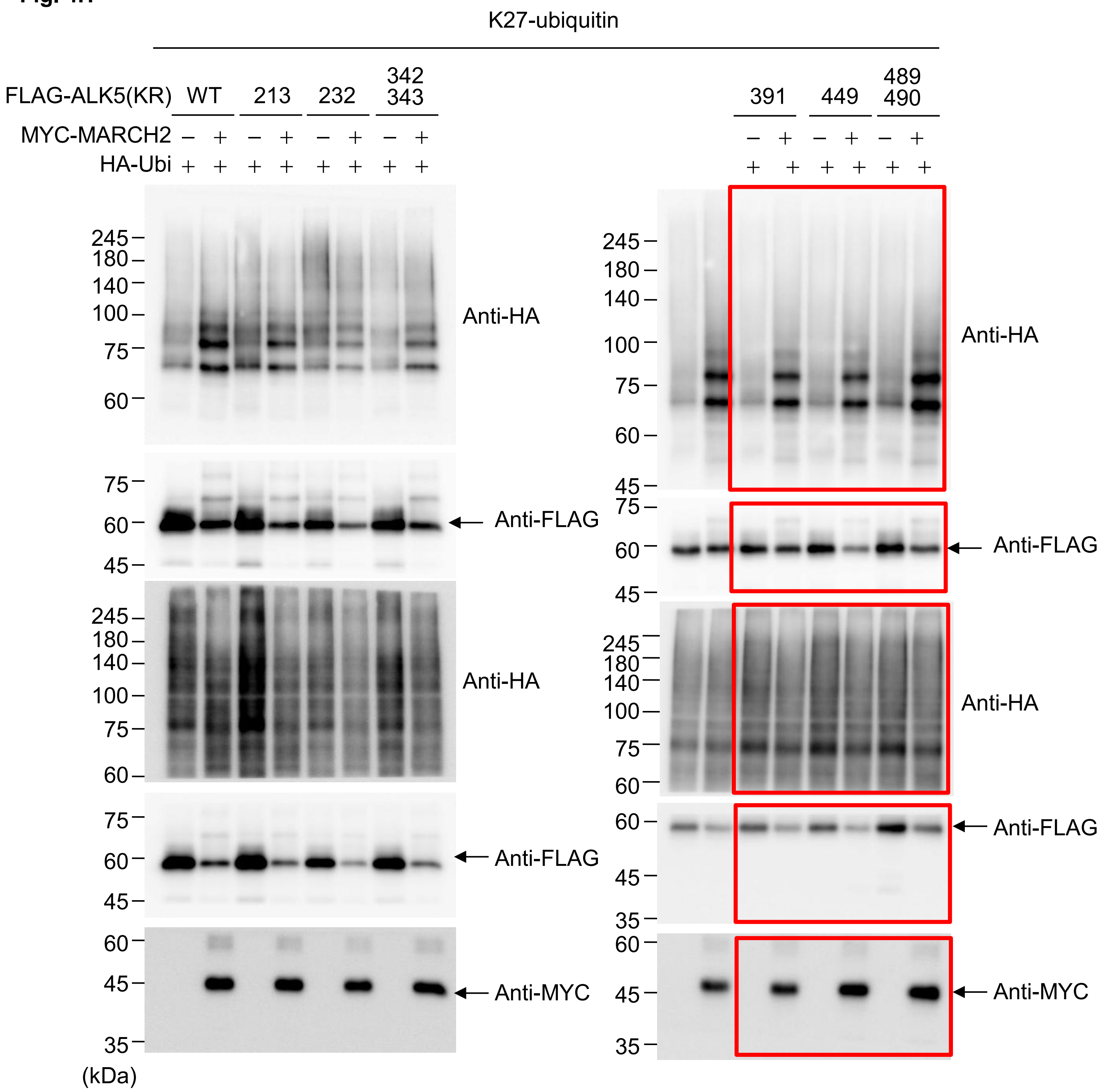
K27-ubiquitin
FLAG-ALK5(KR)
WT
213
232
342 343
391
449
489 490
MYC-MARCH2
HA-Ubi
Anti-HA
Anti-FLAG
Anti-MYC
245
180
140
100
75
60
45
35
(kDa)

Fig. 4H

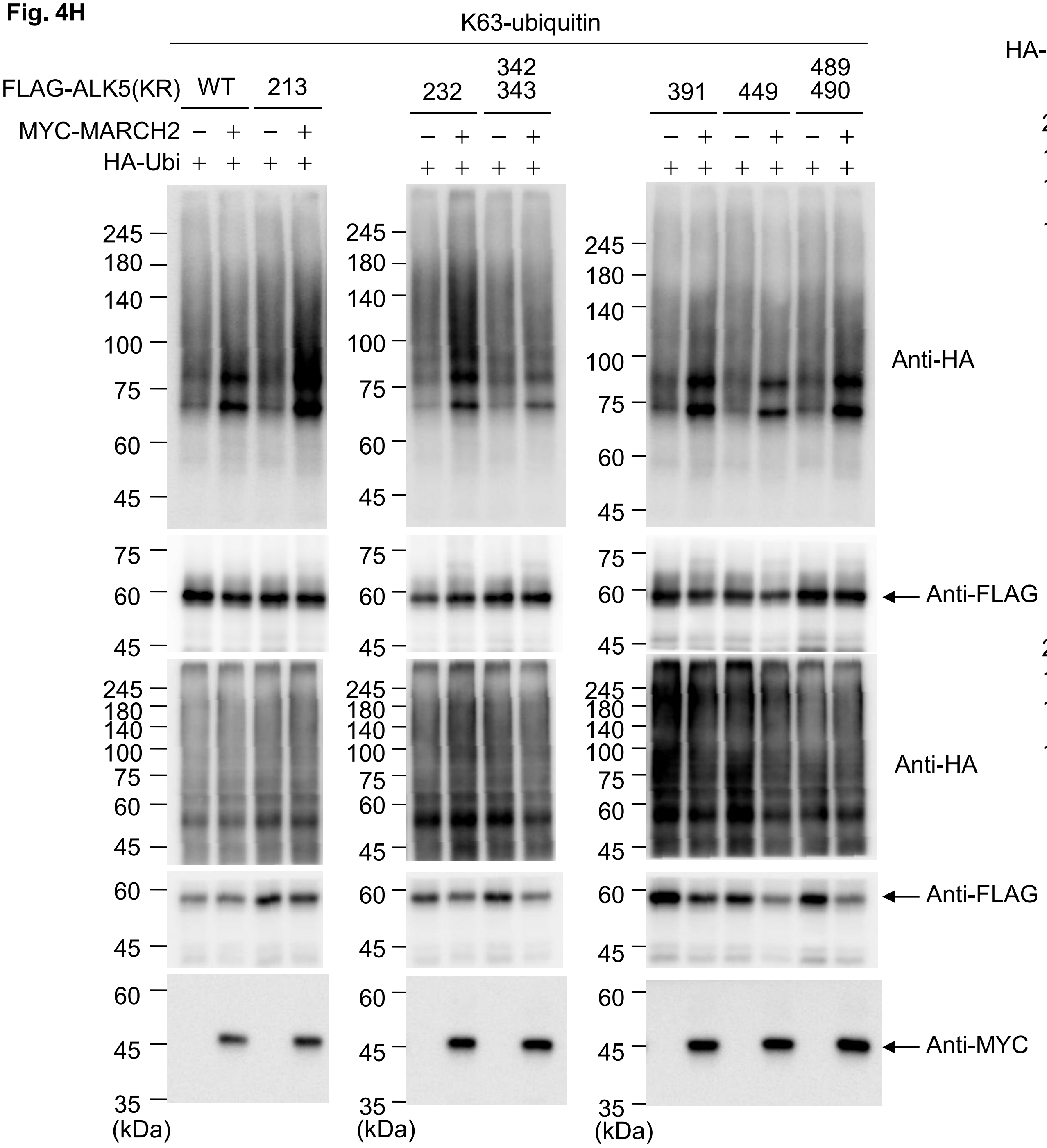
K63-ubiquitin
FLAG-ALK5(KR) WT 213 232 342 343 391 449 489 490
MYC-MARCH2 – + – + – + – + – + – + – +
HA-Ubi + + + + + + + + + + + + + +
245 180 140 100 75 60 45
Anti-HA
75 60 45
Anti-FLAG
245 180 140 100 75 60 45
Anti-HA
60 45
Anti-FLAG
60 45 35
Anti-MYC
(kDa)

Fig. 4I

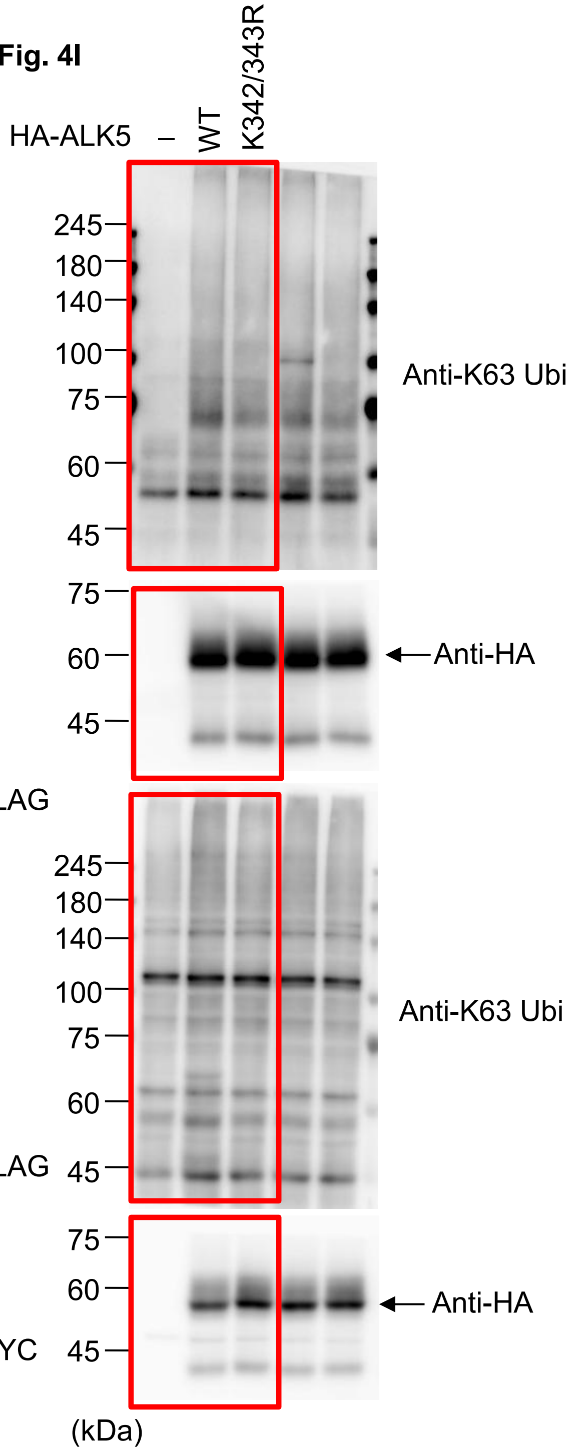
HA-ALK5 – WT K342/343R
245 180 140 100 75 60 45
Anti-K63 Ubi
75 60 45
Anti-HA
245 180 140 100 75 60 45
Anti-K63 Ubi
75 60 45
Anti-HA
(kDa)

Fig. 5A

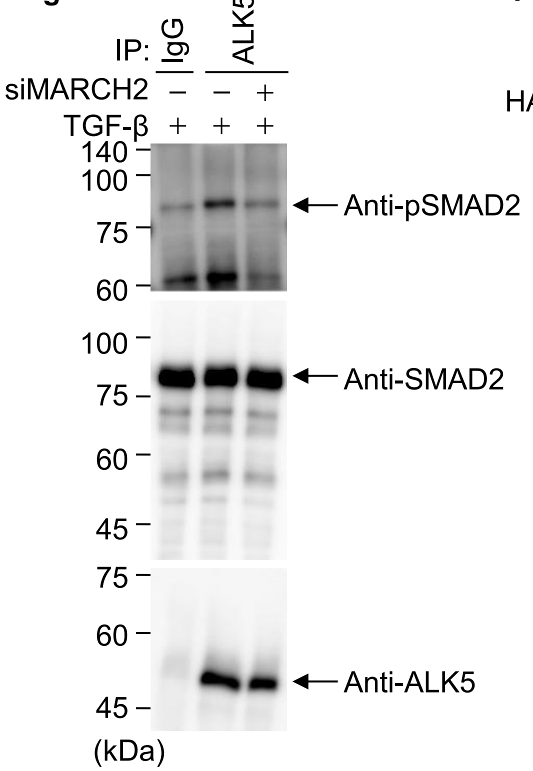

Fig. 5B

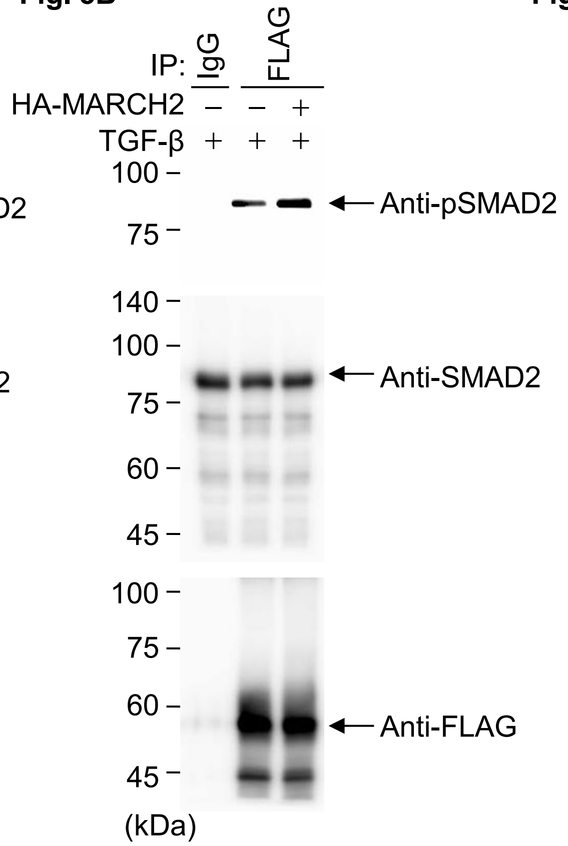

Fig. 5C

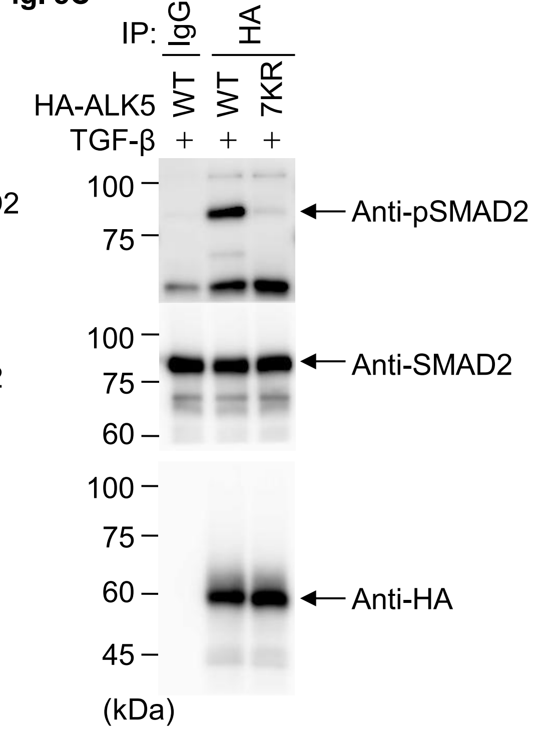

Fig. 5D

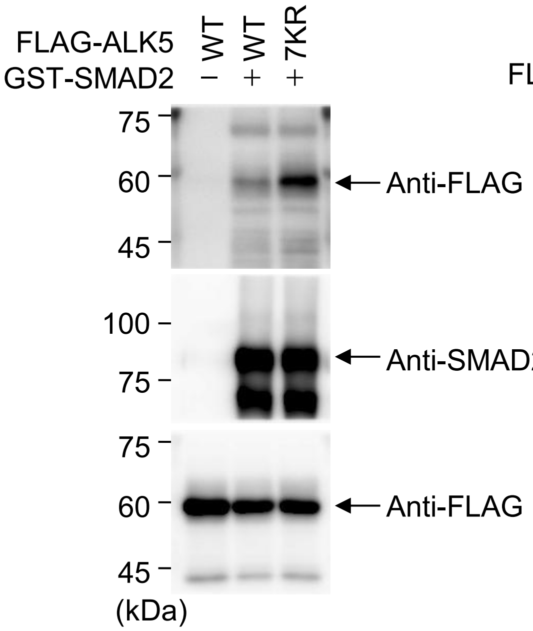

Fig. 5E

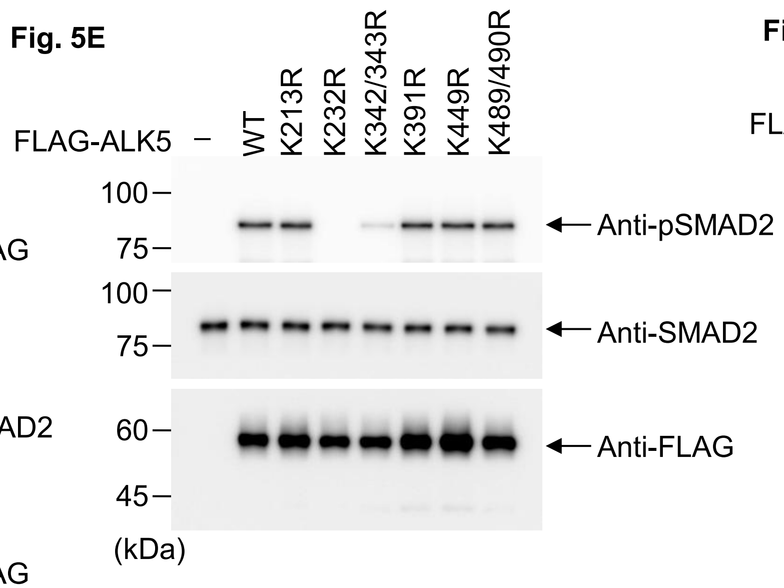

Fig. 5F

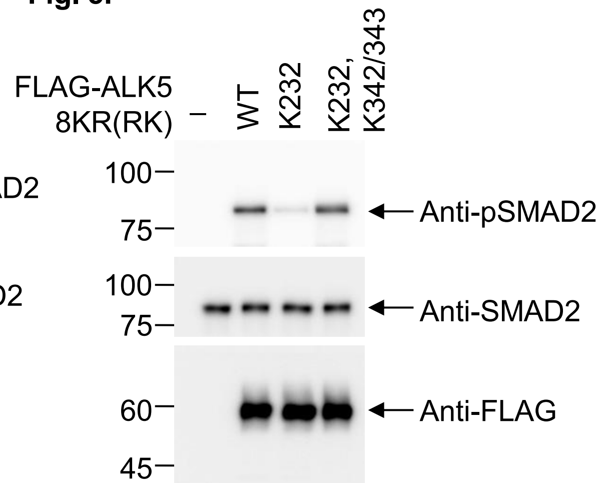

Fig. 5G

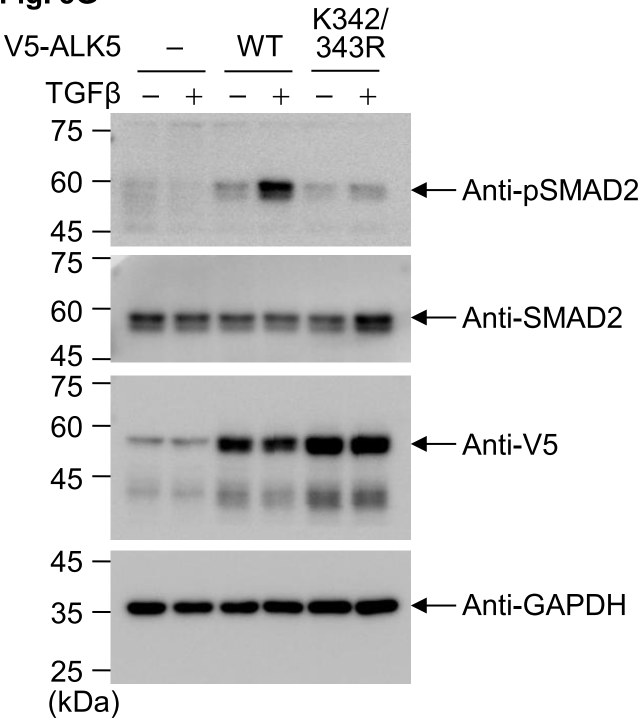
V5-ALK5
–
WT
K342/343R
TGFβ
– + – + – +
75
60
45
Anti-pSMAD2
75
60
45
Anti-SMAD2
75
60
45
Anti-V5
45
35
25
Anti-GAPDH
(kDa)

Fig. 5H

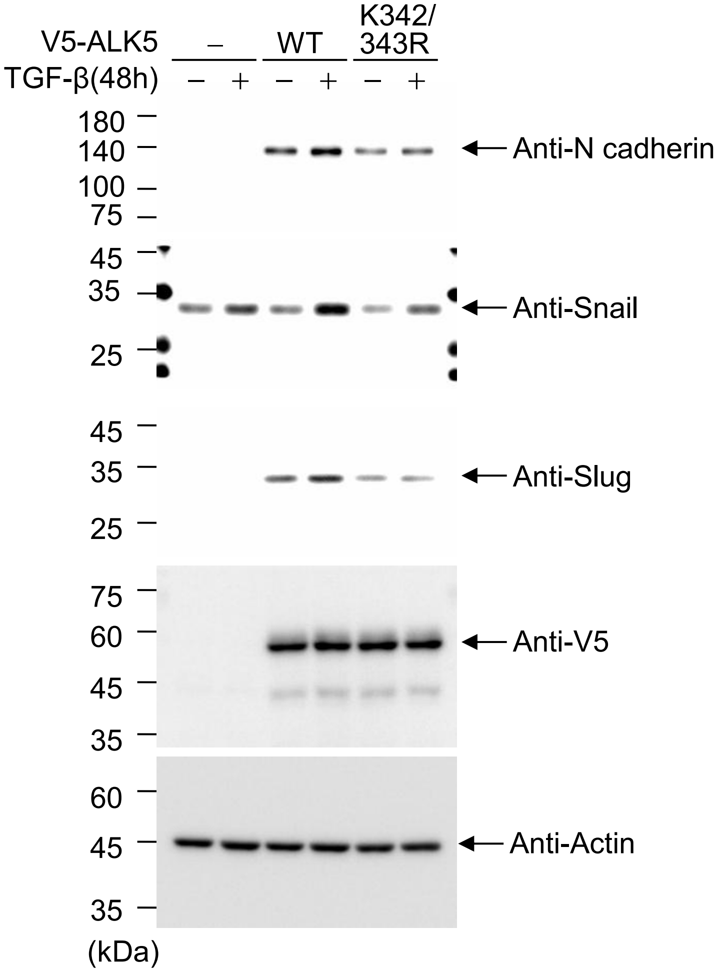
V5-ALK5
–
WT
K342/343R
TGF-β(48h)
– + – + – +
180
140
100
75
Anti-N cadherin
45
35
25
Anti-Snail
45
35
25
Anti-Slug
75
60
45
35
Anti-V5
60
45
35
Anti-Actin
(kDa)

Fig. S1A

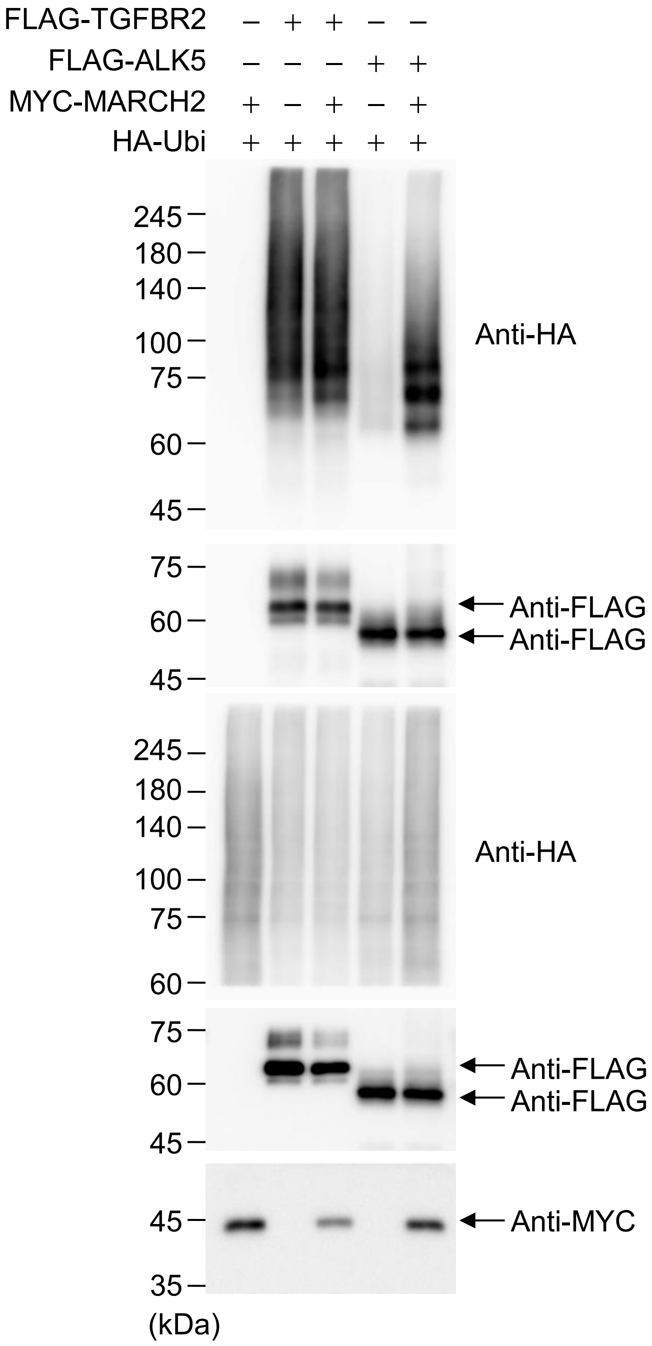

Fig. S1C

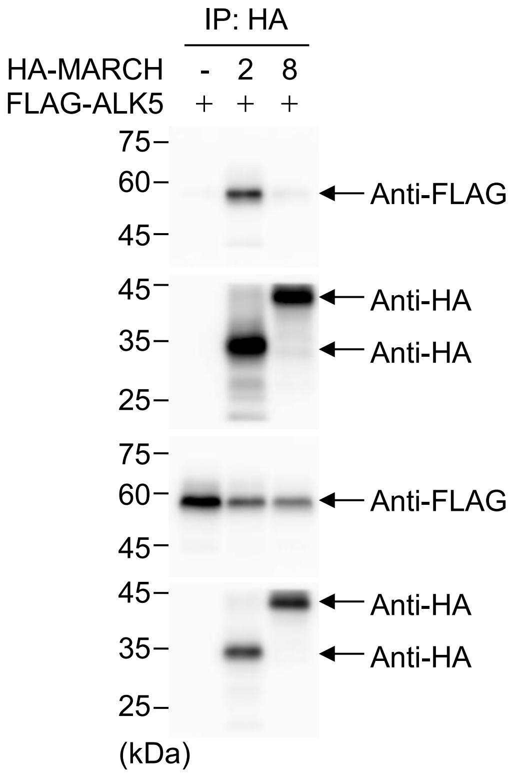

Fig. S3A

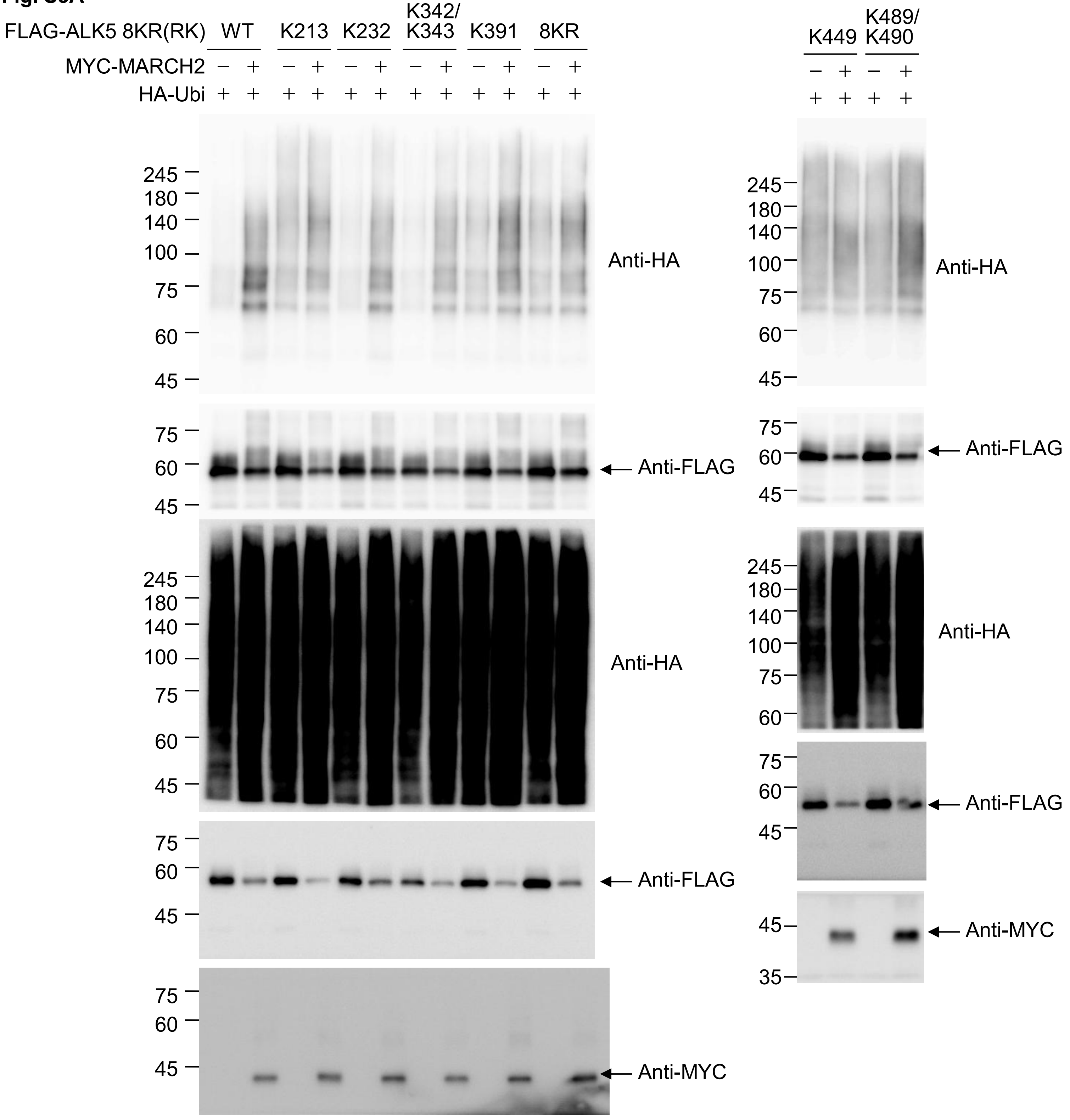
FLAG-ALK5 8KR(RK)
WT
K213
K232
K342/
K343
K391
8KR
K449
K489/
K490
MYC-MARCH2
HA-Ubi
245
180
140
100
75
60
45
35
Anti-HA
Anti-FLAG
Anti-MYC

Fig. S3B

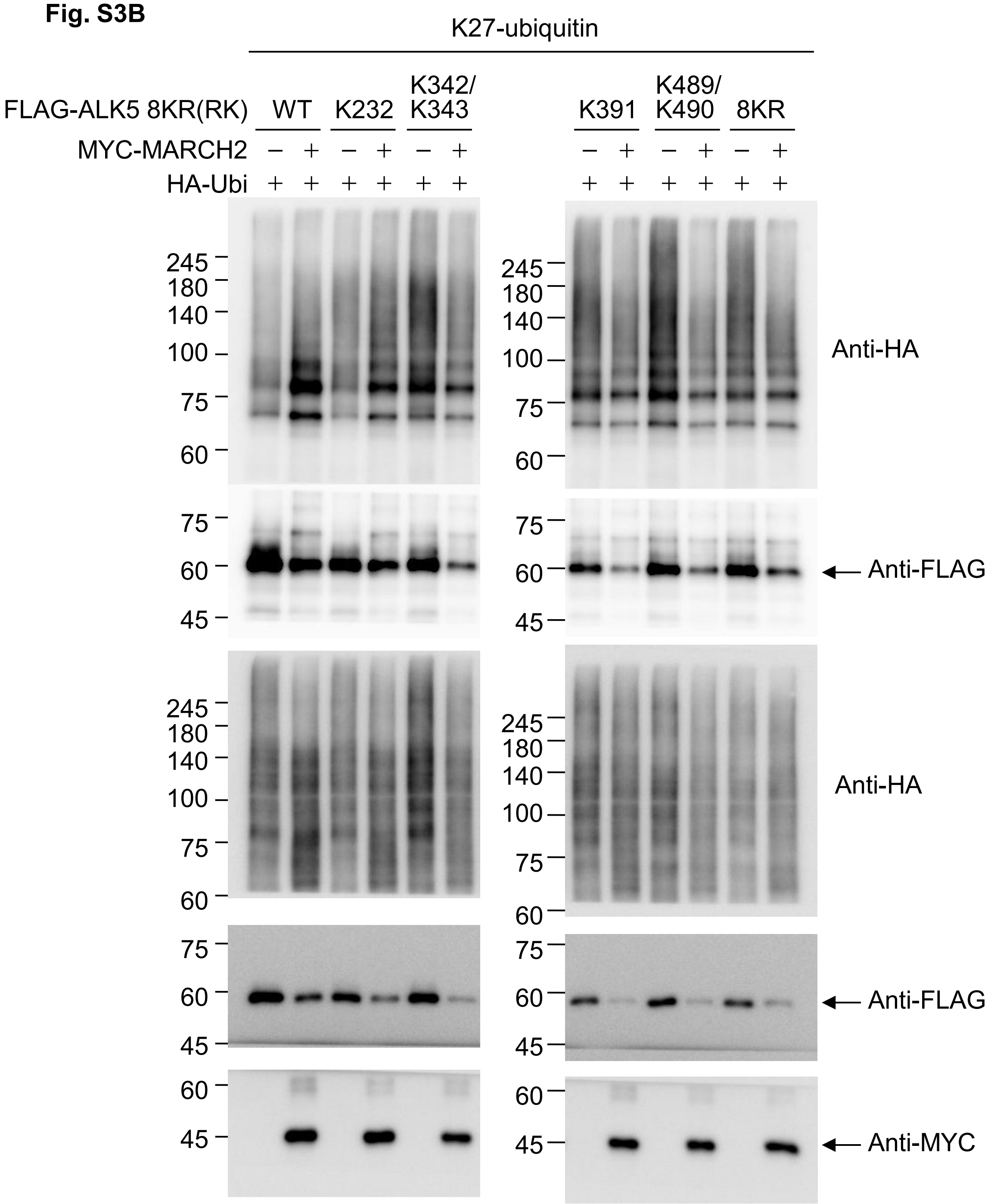
K27-ubiquitin
FLAG-ALK5 8KR(RK)
WT
K232
K342/
K343
K391
K489/
K490
8KR
MYC-MARCH2
HA-Ubi
– + – + – + – + – + – +
+ + + + + + + + + + + +
245
180
140
100
75
60
45
Anti-HA
Anti-FLAG
Anti-MYC

Fig. S3B

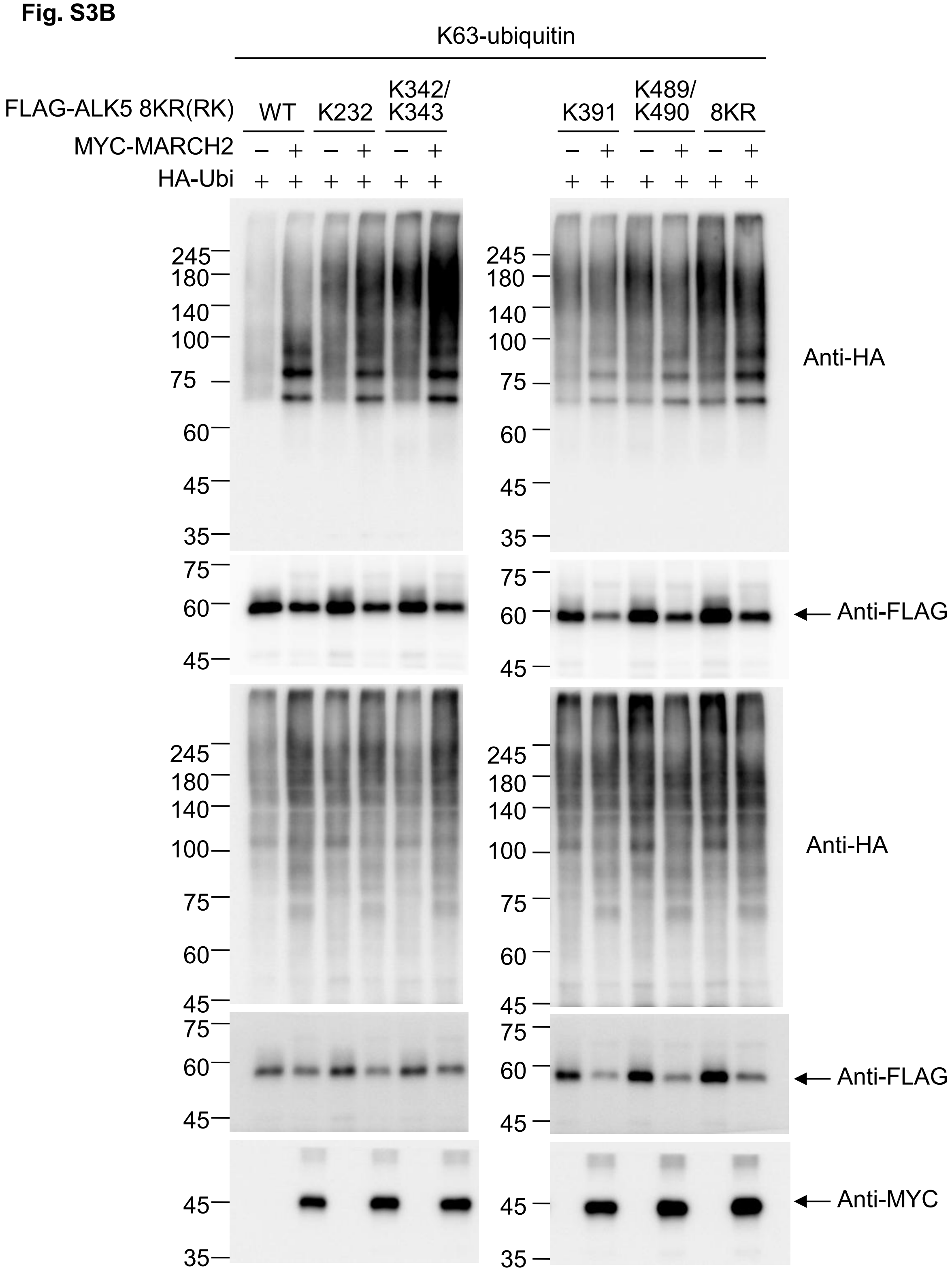
K63-ubiquitin
FLAG-ALK5 8KR(RK)
WT
K232
K342/
K343
K391
K489/
K490
8KR
MYC-MARCH2
HA-Ubi
245
180
140
100
75
60
45
35
Anti-HA
Anti-FLAG
Anti-MYC

Fig. S4A

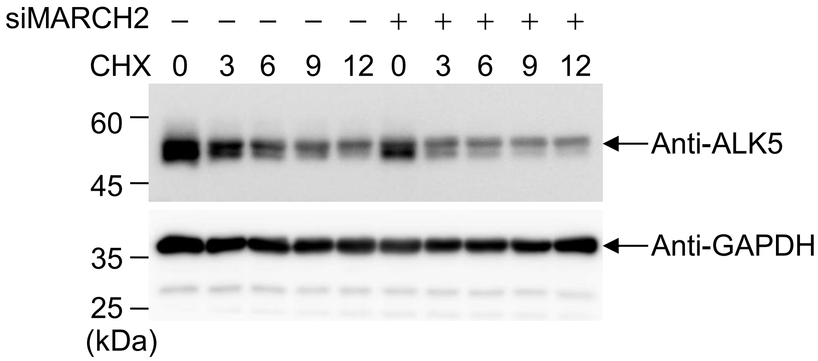

Fig. S4B

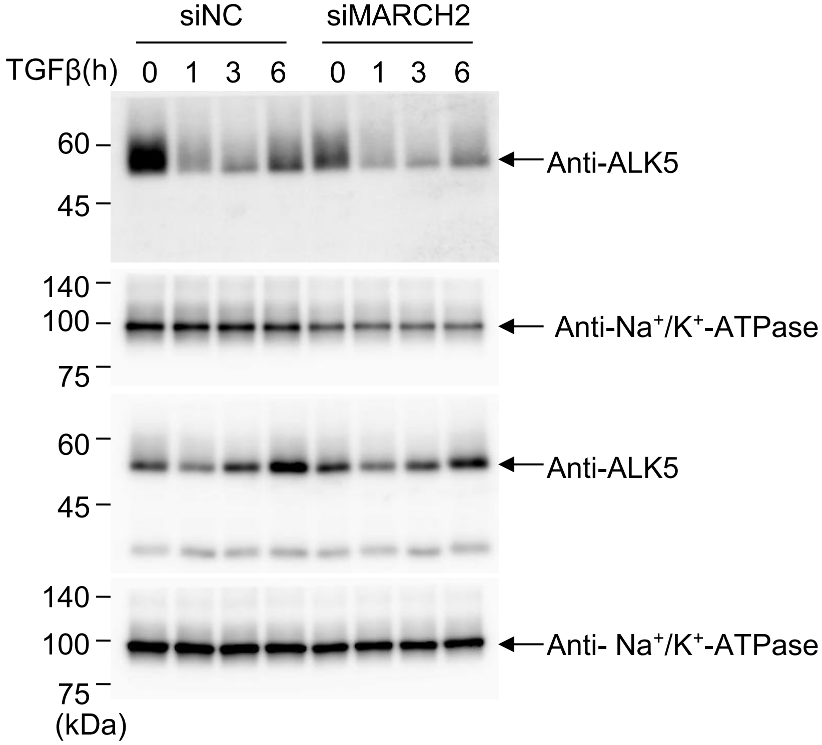

Fig. S5A

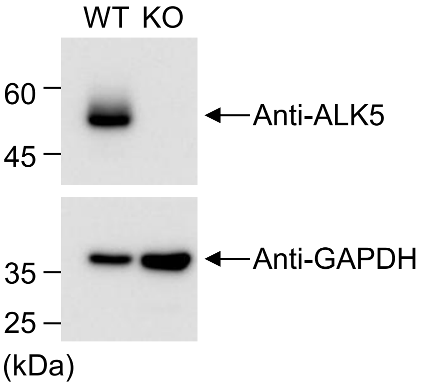

Fig. S5B

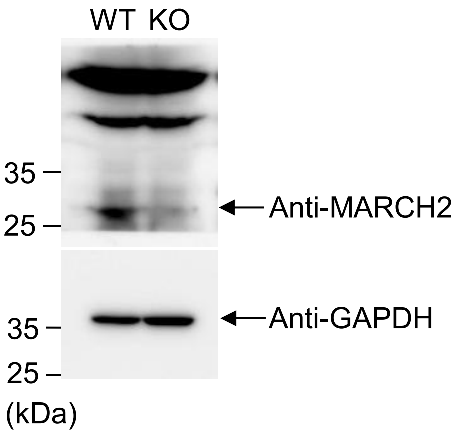

Supplement: Supplementary file 3 — Uncropped WB image [file 41419_2025_8145_MOESM3_ESM.pdf]
